# Association of Placenta Previa With Severe Maternal Morbidity Among Patients With Placenta Accreta Spectrum Disorder

**DOI:** 10.1001/jamanetworkopen.2022.28002

**Published:** 2022-08-22

**Authors:** Xueyan Han, Zhirong Guo, Xinrui Yang, Huixia Yang, Jingmei Ma

**Affiliations:** 1 Department of Medical Statistics, Peking University First Hospital, Beijing, China; 2 Department of Obstetrics and Gynecology, Peking University First Hospital, Beijing, China; 3 Beijing Key Laboratory of Maternal Fetal Medicine of Gestational Diabetes Mellitus, Peking University First Hospital, Beijing, China

## Abstract

**Importance:**

Placenta previa is widely acknowledged as a risk factor for placenta accreta spectrum (PAS) disorders, which are severe maternal complications; however, data are limited regarding whether placenta previa is associated with a higher risk of worse maternal outcomes among patients with PAS disorders.

**Objective:**

To examine the association between placenta previa and the risk of severe maternal morbidities (SMMs) and higher resource use among patients with PAS disorders.

**Design, Setting, and Participants:**

This retrospective cohort study extracted records of 3793 patients with PAS diagnosis and delivery indicators between October 1, 2015, and December 31, 2019, from the US National Inpatient Sample database.

**Exposures:**

Placenta previa.

**Main Outcomes and Measures:**

Data on 21 Centers for Disease Control and Prevention–defined SMMs and 25 study-defined surgical morbidities associated with PAS were extracted. Six surgical procedures (cystoscopy, intra-arterial balloon occlusion, cesarean delivery, hysterectomy, cystectomy, and oophorectomy), hospital length of stay, and inpatient costs were compared. Multivariable Poisson regression models built in the generalized estimating equation framework were used.

**Results:**

Among 3793 patients with PAS (median [IQR] age at admission, 33 [29-37] years), 621 women (16.4%) were Black, 765 (20.2%) were Hispanic, 1779 (46.9%) were White, 441 (11.6%) were of other races and/or ethnicities (47 [1.2%] were American Indian, 220 [5.8%] were Asian or Pacific Islander, and 174 [4.6%] were of multiple or other races and/or ethnicities), and 187 (4.9%) were of unknown race and ethnicity. A total of 1323 patients (34.9%) had placenta previa and 2470 patients (65.1%) did not; of those with placenta previa, 405 patients (30.6%) had invasive PAS. Patients with vs without placenta previa had a significantly higher rate and risk of any SMM (935 women [70.7%] vs 1087 women [44.0%]; *P* < .001; adjusted risk ratio [aRR], 1.19; 95% CI, 1.12-1.27) and any surgical morbidity (1170 women [88.4%] vs 1667 women [67.5%]; *P* < .001; aRR, 1.18; 95% CI, 1.13-1.23). With regard to specific outcomes, those with vs without placenta previa had a significantly higher rate of peripartum hemorrhage (878 patients [66.4%] vs 1217 patients [49.3%]; *P* < .001), blood product transfusion (413 patients [31.2%] vs 610 patients [24.7%]; *P* < .001), shock (83 patients [6.3%] vs 108 patients [4.4%]; *P* = .01), disseminated intravascular coagulation or other coagulopathy (77 patients [5.8%] vs 105 patients [4.3%]; *P* = .04), and urinary tract injury (44 patients [3.3%] vs 41 patients [1.7%]; *P* = .002). Patients with vs without placenta previa were more likely to undergo cesarean delivery (1292 patients [97.7%] vs 1787 patients [72.3%]; *P* < .001), hysterectomy (786 patients [59.4%] vs 689 patients [27.9%]; *P* < .001), cystoscopy (301 patients [22.8%] vs 203 patients [8.2%]; *P* < .001), cystectomy (157 patients [11.9%] vs 98 patients [4.0%]; *P* < .001), and intra-arterial balloon occlusion (121 patients [9.1%] vs 77 patients [3.1%]; *P* < .001) and to have significantly longer hospital length of stay (median [IQR], 5 [4-11] days vs 3 [3-5] days; *P* < .001) and total inpatient costs (median [IQR], $17 496 [$10 863-$30 619] vs $9728 [$6130-$16 790]; *P* < .001). Hypertensive disorder of pregnancy was associated with a decreased risk of placenta previa (aRR, 0.67; 95% CI, 0.46-0.96) among patients with PAS.

**Conclusions and Relevance:**

In this study, placenta previa was associated with an increased risk of maternal and surgical morbidities and higher resource use among women with PAS. These findings suggest that interventions to alleviate maternal and surgical morbidities are especially needed for patients with placenta previa–complicated PAS disorders.

## Key Points

**Question:**

Is placenta previa associated with an increased risk of severe maternal outcomes among patients with placenta accreta spectrum (PAS) disorders?

**Findings:**

In this cohort study of 3793 patients with PAS disorders, placenta previa was associated with a higher risk of severe maternal morbidities and surgical morbidities, increased use of surgical procedures (including intra-arterial balloon occlusion, cystoscopy, cesarean delivery, hysterectomy, and cystectomy), longer hospital stays, and higher inpatient costs.

**Meaning:**

This study found that patients with PAS disorders who experience placenta previa had an increased risk of worse maternal outcomes and higher resource use, suggesting that care routines and procedures that alleviate maternal and surgical morbidities among these patients are needed.

## Teaser

This cohort study assesses the association between placenta previa and the risk of severe maternal morbidities and higher resource use among patients with placenta accreta spectrum disorders in the US.

## Introduction

Placenta accreta spectrum (PAS) disorders are severe maternal complications characterized by abnormal adherence of the placental trophoblast to the uterine myometrium.^1^ Although the prevalence of PAS has been generally lower than 1%,^2,3^ data from a nationally representative all-payer database from 2015 to 2017 revealed that PAS disorders increased approximately 2% every 3 months among US patients undergoing cesarean delivery.^4^ According to current estimation, the incidence rate of PAS may increase to 1 in 200 women undergoing cesarean delivery by 2025.^4^ Because PAS can increase the risk of catastrophic hemorrhage, hysterectomy, organ damage, consumptive coagulopathy, and maternal death,^2,4,5,6,7,8,9,10,11,12,13^ antenatal diagnosis and multidisciplinary management of PAS are important.^5,10,13,14,15,16,17^

Placenta previa is a major risk factor for PAS,^18,19^ and placenta previa itself is a severe pregnancy complication that can lead to substantial postpartum hemorrhage and organ injuries.^20^ According to a cohort study of 605 362 deliveries in Nordic countries, the estimated prevalence of PAS among those with placenta previa (41.8 cases per thousand) was higher than that of all pregnancies (0.3 cases per thousand) or pregnancies with previous cesarean delivery (8.8 cases per thousand among those with 3 previous cesarean deliveries).^19,21^ Therefore, multiple guidelines^16,22,23^ have recommended that patients with a diagnosis of placenta previa be screened vigorously for placental adherence using multimodal ultrasonographic and magnetic resonance imaging. However, placenta previa may not only be a risk factor for PAS ; placenta previa may also be associated with an increased risk of severe maternal outcomes among patients diagnosed with PAS.

Because both placenta previa and PAS are independently associated with substantial morbidity and mortality,^4,24,25,26,27^ pregnant patients with both PAS and placenta previa complications could experience a compounded risk of maternal morbidities and have worse outcomes than patients who have PAS but not placenta previa. However, there are contradictory results regarding this issue. A study by Mulla et al^28^ involving 105 patients with PAS found that those with coexisting placenta previa had substantially higher median estimated perinatal blood loss volume than those without placenta previa (3500 mL vs 1200 mL; *P* < .001). Heading et al^29^ also found that, among 134 patients with PAS, those with placenta previa had substantially higher estimated blood loss volume. However, Carusi et al^30^ analyzed data from 351 patients with PAS who underwent hysterectomy and found no statistically significant difference in estimated blood loss volume and the number of red blood cell transfusion units among patients with PAS who did vs did not have placenta previa.

More evidence on whether placenta previa is associated with a higher risk of worse maternal outcomes in patients with PAS is needed, especially data from studies with multicenter designs and large samples. Previous studies^28,29^ were conducted in a single center with relatively small samples and may have had insufficient statistical power that prevented them from adjusting for a larger number of known risk factors associated with severe maternal morbidities (SMMs). It can also be difficult to observe outcomes, such as acute kidney injury, urinary tract injury, disseminated intravascular coagulation, and shock, in single-center studies because these events are rare. Assessment and comparison of the use of surgical procedures and health care resources are also needed so that a more comprehensive understanding of maternal outcomes among patients who have PAS with and without placenta previa can be provided.

This cohort study aimed to compare pregnancy characteristics, surgical procedure use, and adverse maternal outcomes among patients with PAS disorders with and without coexisting placenta previa using the US National Inpatient Sample (NIS) database.^31^ The results of this study may provide data regarding screening, early detection, and pre- and postnatal care of patients with PAS and further understanding about the association between placenta previa and the risk of severe maternal morbidities and adverse outcomes among these patients.

## Methods

The Science Research Ethics Committee of the Peking University First Hospital in Beijing, China, approved this retrospective cohort study and determined that it was exempt from human participant research review. There was no patient or public involvement in this study. The study followed the Strengthening the Reporting of Observational Studies in Epidemiology (STROBE) guideline for cohort studies.^32^

### Study Population

This retrospective cohort study used data from the NIS database of the Agency for Healthcare Research and Quality. The NIS is a deidentified all-payer inpatient database capturing approximately 20% of US community hospital admissions. This study included NIS data from October 1, 2015, to December 31, 2019. Data were extracted based on diagnosis or procedure codes.

### Eligibility Criteria

Admission records with the following criteria were included: (1) women with a diagnosis code for PAS (O432) from the *International Statistical Classification of Diseases and Related Health Problems, Tenth Revision *(*ICD-10*) and (2) women who had delivery indicators per NIS criteria.^33^ Those who were discharged against medical advice, had missing values for important variables (ie, age), or had a hospital length of stay (LOS) of 0 days or more than 60 days were excluded. The flowchart of data inclusion and exclusion is provided in the Figure.

**Figure.**
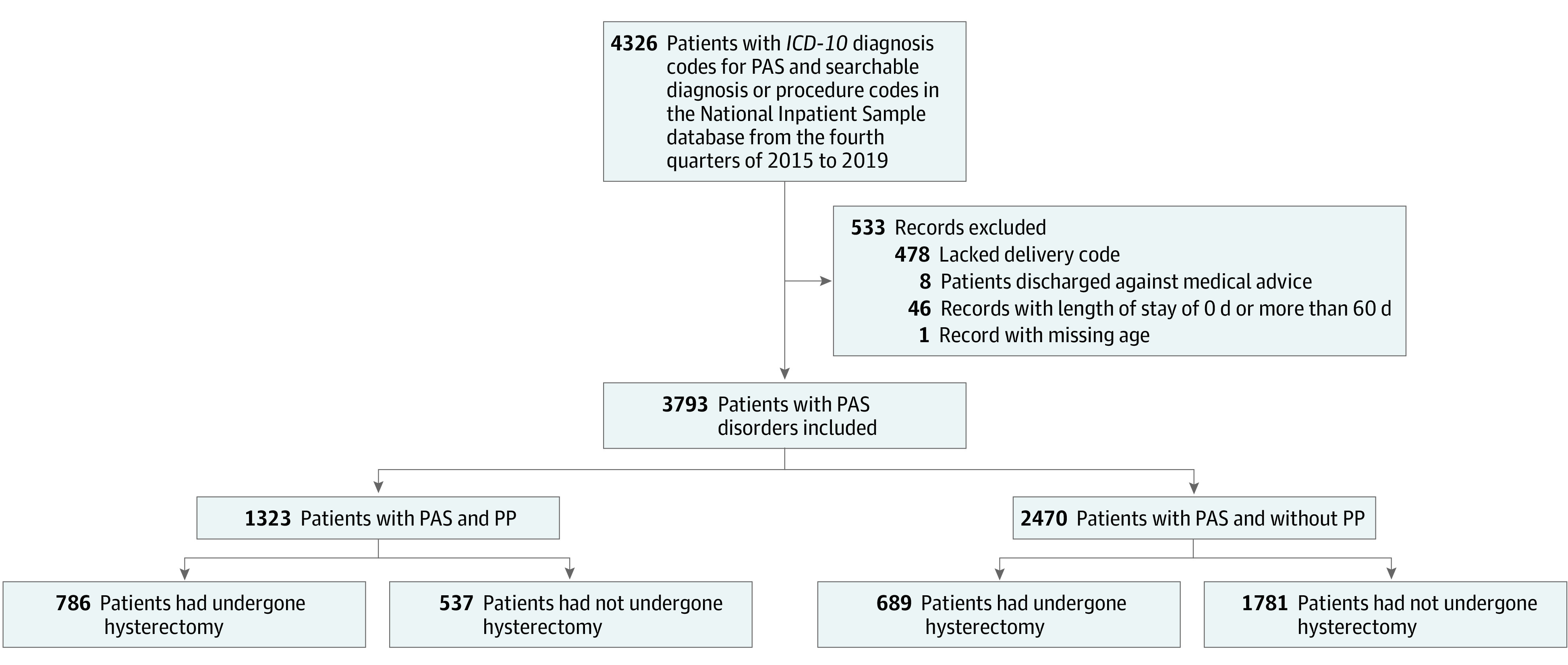
Patient Flowchart ICD-10 indicates *International Statistical Classification of Diseases and Related Health Problems, Tenth Revision*; PAS, placenta accreta spectrum; PP, placenta previa.

### Outcomes

We used the SMM list developed by the US Centers for Disease Control and Prevention^34^ and a list of surgical morbidities used in previous studies.^4,35,36^ The SMM list comprised 21 severe complications of labor and delivery, including blood transfusion, hysterectomy, disseminated intravascular coagulation, heart failure, sepsis, and shock (eTable 1 in the Supplement). The list of surgical morbidities was developed by Matsuzaki et al^4^ and included 25 morbidities (eTable 2 in the Supplement). We also compared the use of 6 surgical procedures (cesarean delivery, hysterectomy, oophorectomy, cystoscopy, intra-arterial balloon occlusion,^37^ and urinary system repair and cystectomy) and 6 specific outcomes derived from the surgical morbidity and SMM lists (eTable 3 in the Supplement). We also extracted hospital LOS, postoperative hospital LOS (for those who underwent cesarean delivery), total charges (charges converted to 2019 US dollars using annual consumer price indexes), and total costs (charges converted to 2019 US dollars and adjusted using the Healthcare Cost and Utilization Project cost-to-charges ratio^31^).

### Exposure

The primary exposure in this study was placenta previa (*ICD-10* code O44) (eTable 4 in the Supplement). Patient demographic information, pregnancy characteristics, PAS severity, and comorbidities were also extracted (eTables 5-7 in the Supplement).^38,39,40,41^ Data on race and ethnicity were originally provided by the partner organizations of Healthcare Cost and Utilization, Agency for Healthcare Research and Quality,^42^ and then extracted from the NIS database. This information was included in the model as one of the 997 patient-level adjustment variables.

### Statistical Analysis

For descriptive analysis, continuous variables were reported as means with SDs or medians with IQRs. Independent sample *t* tests or rank-sum tests were used for group comparisons. Categorical data were expressed as counts with percentages, and χ^2^ tests or Fisher exact tests were used for group comparisons. Because the study did not aim to obtain national estimates, the NIS database was used as an unweighted data set, and we did not account for the database’s stratified sampling design.^43^

To evaluate the association between placenta previa and the risk of clinical outcomes, we used multivariable Poisson regression analysis with robust error variance built in a generalized estimating equation framework to control for the clustering effect of hospitals (exchangeable or independent correlation structures were used in the generalized estimating equation models).^4^ Continuous outcomes (hospital LOS and adjusted cost) were dichotomized by setting the cutoff value as a rounded number near the 75th percentile.^44^

For each outcome measure, we first applied a predefined adjustment model^45,46,47^ to assess the adjusted association of placenta previa with the risk of PAS outcomes. The following factors were included along with placenta previa in the predefined model: age, year, race and ethnicity, obesity status, Charlson Comorbidity Index score, PAS severity, previous cesarean delivery, multiparity, gestational weeks, the use of assisted reproductive technology, multiple gestation, hospital bed size, and hospital teaching status.

A series of sensitivity analyses were conducted to assess the robustness of the findings. To test for the sensitivity of the outcome measures, surgical morbidity and SMM outcomes excluding blood transfusion and/or hysterectomy were used as dependent variables. To evaluate associations between placenta previa and maternal outcomes in patients with PAS of different severities, analyses were conducted among those who underwent hysterectomy (n = 1475), those with invasive PAS (placenta increta or placenta percreta) (n = 768), and those who underwent cesarean delivery (n = 3079). Details on the regression model designs are shown in eTable 8 in the Supplement.

Missing cases were grouped in categories based on applicable variables.^4^ Statistical tests were all based on 2-tailed hypotheses. The coefficients of Poisson regression models were reported as risk ratios (RRs) or adjusted RRs (aRRs)^48,49,50^ with 95% CIs. Statistical significance was set at 2-tailed *P* < .05. All analyses were conducted using Stata software, version 15.0 (StataCorp LLC).

## Results

### Patient Characteristics

A total of 3793 women with PAS (median [IQR] age at admission, 33 [29-37] years) were included; 621 women (16.4%) were Black, 765 (20.2%) were Hispanic, 1779 (46.9%) were White, 441 (11.6%) were of other races and/or ethnicities (47 [1.2%] were American Indian, 220 [5.8%] were Asian or Pacific Islander, and 174 [4.6%] were of multiple or other races and/or ethnicities), and 187 (4.9%) were of unknown race and ethnicity. Overall, 1323 patients (34.9%) had placenta previa, and 2470 (65.1%) did not (Table 1). Among those with placenta previa, 405 patients (30.6%) had invasive PAS, 1212 (91.6%) had complete placenta previa, and 659 (49.8%) had placenta previa with hemorrhage (eTable 9 in the Supplement).

**Table 1.** Sociodemographic and Clinical Characteristics of Patients With PAS Disorder With and Without Placenta Previa

Characteristic	Patients, No. (%)			*P* value
	Total (n = 3793)	Without placenta previa (n = 2470)	With placenta previa (n = 1323)	
**Demographic**				
Year				
2015 (Fourth quarter)	225 (5.9)	145 (5.9)	80 (6.0)	.88
2016	851 (22.4)	563 (22.8)	288 (21.8)	
2017	875 (23.1)	576 (23.3)	299 (22.6)	
2018	930 (24.5)	602 (24.4)	328 (24.8)	
2019	912 (24.0)	584 (23.6)	328 (24.8)	
Age at admission, median (IQR), y	33 (29-37)	33 (29-37)	33 (29-37)	.08
Advanced maternal age (≥35 y)	1527 (40.3)	982 (39.8)	545 (41.2)	.39
Race and ethnicity				
Black	621 (16.4)	411 (16.6)	210 (15.9)	<.001
Hispanic	765 (20.2)	432 (17.5)	333 (25.2)	
White	1779 (46.9)	1230 (50.0)	549 (41.5)	
Other^a^	441 (11.6)	269 (10.9)	172 (13.0)	
Unknown	187 (4.9)	128 (5.2)	59 (4.5)	
Elective admission	1940 (51.1)	1323 (53.6)	617 (46.6)	<.001
ED service use^b^	407 (10.7)	249 (10.1)	158 (11.9)	.08
Insurance status				
Medicare or Medicaid	1727 (45.5)	1054 (42.7)	673 (50.9)	<.001
Private	1856 (48.9)	1278 (51.7)	578 (43.7)	
Other	210 (5.5)	137 (5.5)	71 (5.4)	
Hospital bed size				
Small	479 (12.6)	364 (14.7)	115 (8.7)	<.001
Medium	925 (24.4)	662 (26.8)	263 (19.9)	
Large	2389 (63.0)	1444 (58.5)	945 (71.4)	
Hospital location and teaching status				
Rural	215 (5.7)	187 (7.6)	28 (2.1)	<.001
Urban nonteaching	511 (13.5)	388 (15.7)	123 (9.3)	
Urban teaching	3067 (80.9)	1895 (76.7)	1172 (88.6)	
Hospital region				
Northeast	689 (18.2)	434 (17.6)	255 (19.3)	.46
Midwest	819 (21.6)	545 (22.1)	274 (20.7)	
South	1332 (35.1)	877 (35.5)	455 (34.4)	
West	953 (25.1)	614 (24.9)	339 (25.6)	
Pregnancy history				
Grand multiparity	40 (1.1)	27 (1.1)	13 (1.0)	.75
Previous cesarean delivery	2166 (57.1)	1211 (49.0)	955 (72.2)	<.001
**Current pregnancy status**				
ART	51 (1.3)	38 (1.5)	13 (1.0)	.16
Charlson Comorbidity Index score				
0	3358 (88.5)	2196 (88.9)	1162 (87.8)	.45
1	397 (10.5)	247 (10.0)	150 (11.3)	
2	28 (0.7)	19 (0.8)	NR^c^	
≥3	10 (0.3)	NR^c^	NR^c^	
Elixhauser comorbidity score				
0	2154 (56.8)	1500 (60.7)	654 (49.4)	<.001
1	1035 (27.3)	629 (25.5)	406 (30.7)	
2	434 (11.4)	252 (10.2)	182 (13.8)	
≥3	170 (4.5)	89 (3.6)	81 (6.1)	
Obesity status^d^				
Class 0 (Nonobesity)	3199 (84.3)	2130 (86.2)	1069 (80.8)	<.001
Class 1-2	332 (8.8)	180 (7.3)	152 (11.5)	
Class 3	262 (6.9)	160 (6.5)	102 (7.7)	
Tobacco use	327 (8.6)	209 (8.5)	118 (8.9)	.63
PAS type				
Accreta	3025 (79.8)	2107 (85.3)	918 (69.4)	<.001
Increta	339 (8.9)	158 (6.4)	181 (13.7)	
Percreta	429 (11.3)	205 (8.3)	224 (16.9)	
Trimester of PAS diagnosis				
1	NR^c^	NR^c^	NR^c^	.11
2	214 (5.6)	123 (5.0)	91 (6.9)	
3	3442 (90.7)	2258 (91.4)	1184 (89.5)	
Unspecified	133 (3.5)	86 (3.5)	47 (3.6)	
Gestation, wk				
<32	478 (12.6)	220 (8.9)	258 (19.5)	<.001
32-33	360 (9.5)	145 (5.9)	215 (16.3)	
34-36	1212 (32.0)	603 (24.4)	609 (46.0)	
>36	1649 (43.5)	1458 (59.0)	191 (14.4)	
Unknown	94 (2.5)	44 (1.8)	50 (3.8)	
Pregnancy complications				
Preterm birth	2465 (65.0)	1275 (51.6)	1190 (89.9)	<.001
Multiple gestation	154 (4.1)	116 (4.7)	38 (2.9)	.007
Gestational diabetes	464 (12.2)	290 (11.7)	174 (13.2)	.21
HDP	271 (7.1)	221 (8.9)	50 (3.8)	<.001
Breech presentation	543 (14.3)	310 (12.6)	233 (17.6)	<.001
PPROM	218 (5.7)	160 (6.5)	58 (4.4)	.008
FGR	203 (5.4)	150 (6.1)	53 (4.0)	.007

Abbreviations: ART, assisted reproductive technology; ED, emergency department; FGR, fetal growth restriction; HDP, hypertensive disorder of pregnancy; NR, not reported; PAS, placenta accreta spectrum; PPROM, preterm premature rupture of membranes.

^a^ Other races and/or ethnicities (in the entire sample) included American Indian (47 patients [1.2%]), Asian or Pacific Islander (220 patients [5.8%]), and multiple or other races and/or ethnicities (174 patients [4.6%]).

^b^ Records with evidence of emergency service use per criteria from the Healthcare Cost and Utilization Project.

^c^ Data were suppressed for categories with fewer than 10 patients per requirements of the Healthcare Cost and Utilization Project.

^d^ Class 0 (nonobesity) was defined as body mass index (BMI; calculated as weight in kilograms divided by height in meters squared) of <30, class 1 as BMI of 30 to <35, class 2 as BMI of 35 to <40, and class 3 as BMI of ≥40.

Patients with vs without placenta previa were significantly more likely to have nonelective admission (706 patients [53.4%] vs 1147 patients [46.4%]; *P* < .001), have Medicare or Medicaid insurance (673 patients [50.9%] vs 1054 [42.7%]), and be admitted to a large hospital (945 patients [71.4%] vs 1444 patients [58.5%]) or an urban teaching hospital (1172 patients [88.6%] vs 1895 patients [76.7%]). With regard to pregnancy history, the previous cesarean delivery rate among patients with placenta previa was approximately 1.5 times higher than the rate among those without placenta previa (955 patients [72.2%] vs 1211 patients [49.0%]; *P* < .001). Patients with vs without placenta previa were also more likely to have invasive PAS (405 patients [30.6%] vs 363 patients [14.7%]; *P* < .001), higher Elixhauser Comorbidity Index scores (eg, ≥3: 81 patients [6.1%] vs 89 patients [3.6%]), and obesity (eg, class 3 [body mass index ≥40; calculated as weight in kilograms divided by height in meters squared]: 102 patients [7.7%] vs 160 patients [6.5%]). In addition, patients with vs without placenta previa had lower rates of multiple gestation (38 patients [2.9%] vs 116 patients [4.7%]; *P* = .007), hypertensive disorder of pregnancy (50 patients [3.8%] vs 221 patients [8.9%]; *P* < .001), preterm premature rupture of membranes (58 patients [4.4%] vs 160 patients [6.5%]; *P* = .008), and fetal growth restriction (53 patients [4.0%] vs 150 patients [6.1%]; *P* = .007) (Table 1).

### Maternal and Surgical Use Outcomes

Among all patients with PAS, those with vs without placenta previa were significantly more likely to have any surgical morbidity (1170 patients [88.4%] vs 1667 patients [67.5%]; *P* < .001), any SMM (935 patients [70.7%] vs 1087 patients [44.0%]; *P* < .001), hemorrhage (878 patients [66.4%] vs 1217 patients [49.3%]; *P* < .001), blood product transfusion (413 patients [31.2%] vs 610 patients [24.7%]; *P* < .001), shock (83 patients [6.3%] vs 108 patients [4.4%]; *P* = .01), disseminated intravascular coagulation or other coagulopathy (77 patients [5.8%] vs 105 patients [4.3%]; *P* = .04), and urinary tract injury (44 patients [3.3%] vs 41 patients [1.7%]; *P* = .002) and to have longer hospital LOS (median [IQR], 5 [4-11] days vs 3 [3-5] days; *P* < .001) (Table 2). With regard to surgical use, almost all patients with placenta previa underwent cesarean delivery (1292 patients [97.7%] vs 1787 patients [72.3%] without placenta previa; *P* < .001). Those with vs without placenta previa were more likely to undergo hysterectomy (786 patients [59.4%] vs 689 patients [27.9%]; *P* < .001), cystoscopy (301 patients [22.8%] vs 203 patients [8.2%]; *P* < .001), urinary system repair and cystectomy (157 patients [11.9%] vs 98 patients [4.0%]; *P* < .001), and intra-arterial balloon occlusion (121 patients [9.1%] vs 77 patients [3.1%]; *P* < .001). The median overall inpatient charges and costs among patients with placenta previa were almost twice as high as those among patients without placenta previa (total charges: median [IQR], $68 660 [$41 590-$119 022]) vs $36 942 [$22 262-$65 513]; total costs: median [IQR], $17 496 [$10 863-$30 619]) vs $9728 [$6130-$16 790]; *P* < .001 for both comparisons).

**Table 2.** Maternal and Service Use Outcomes in Patients With PAS Disorder With and Without Placenta Previa

Outcome	Patients, No. (%)							
	Total				With hysterectomy			
	Total (n = 3793)	Without placenta previa (n = 2470)	With placenta previa (n = 1323)	*P* value	Total (n = 1475)	Without placenta previa (n = 689)	With placenta previa (n = 786)	*P* value
**Perinatal outcomes**								
Study-defined SM								
Any	2837 (74.8)	1667 (67.5)	1170 (88.4)	<.001	1475 (100)	689 (100)	786 (100)	NA
Excluding transfusion	2759 (72.7)	1610 (65.2)	1149 (86.8)	<.001	1475 (100)	689 (100)	786 (100)	NA
Excluding hysterectomy	2511 (66.2)	1484 (60.1)	1027 (77.6)	<.001	1149 (77.9)	506 (73.4)	643 (81.8)	<.001
Excluding transfusion and/or hysterectomy	2305 (60.8)	1342 (54.3)	963 (72.8)	<.001	1021 (69.2)	421 (61.1)	600 (76.3)	<.001
CDC-defined SMM								
Any	2022 (53.3)	1087 (44.0)	935 (70.7)	<.001	1475 (100)	689 (100)	786 (100)	NA
Excluding transfusion	1621 (42.7)	796 (32.2)	825 (62.4)	<.001	1475 (100)	689 (100)	786 (100)	NA
Excluding hysterectomy	1242 (32.7)	728 (29.5)	514 (38.9)	<.001	695 (47.1)	330 (47.9)	365 (46.4)	.60
Excluding transfusion and/or hysterectomy	394 (10.4)	222 (9.0)	172 (13.0)	<.001	248 (16.8)	115 (16.7)	133 (16.9)	.94
**Specific outcomes**								
Hemorrhage	2095 (55.2)	1217 (49.3)	878 (66.4)	<.001	898 (60.9)	360 (52.2)	538 (68.4)	<.001
Blood product transfusion	1023 (27.0)	610 (24.7)	413 (31.2)	<.001	559 (37.9)	273 (39.6)	286 (36.4)	.22
Shock	191 (5.0)	108 (4.4)	83 (6.3)	.01	124 (8.4)	61 (8.9)	63 (8.0)	.57
Acute kidney injury	43 (1.1)	27 (1.1)	16 (1.2)	.75	29 (2.0)	16 (2.3)	13 (1.7)	.45
DIC or other coagulopathy	182 (4.8)	105 (4.3)	77 (5.8)	.04	116 (7.9)	59 (8.6)	57 (7.3)	.38
Urinary tract injury	85 (2.2)	41 (1.7)	44 (3.3)	.002	65 (4.4)	32 (4.6)	33 (4.2)	.70
**Surgical procedure use**								
Cesarean delivery	3079 (81.2)	1787 (72.3)	1292 (97.7)	<.001	1374 (93.2)	597 (86.6)	777 (98.9)	<.001
Hysterectomy	1475 (38.9)	689 (27.9)	786 (59.4)	<.001	1475 (100)	689 (100)	786 (100)	NA
Oophorectomy	149 (3.9)	82 (3.3)	67 (5.1)	.011	112 (7.6)	58 (8.4)	54 (6.9)	.28
Cystoscopy	504 (13.3)	203 (8.2)	301 (22.8)	<.001	387 (26.2)	148 (21.5)	239 (30.4)	<.001
Urinary system repair and cystectomy	255 (6.7)	98 (4.0)	157 (11.9)	<.001	208 (14.1)	80 (11.6)	128 (16.3)	.01
Intra-arterial balloon occlusion	198 (5.2)	77 (3.1)	121 (9.1)	<.001	110 (7.5)	33 (4.8)	77 (9.8)	<.001
**LOS, median (IQR)**								
Hospital, d	4 (3-6)	3 (3-5)	5 (4-11)	<.001	5 (4-9)	4 (3-6)	6 (4-12)	<.001
After cesarean delivery, d	4 (3-4)	3 (3-4)	4 (3-4)	<.001	4 (3-5)	4 (3-5)	4 (3-5)	.01
**Cost, median (IQR), $**								
Total charges^a^	45 942 (26 000-84 521)	36 942 (22 262-65 513)	68 660 (41 590-119 022)	<.001	72 669 (45 315-126 578)	64 578 (40 292-109 169)	81 814 (50 629-144 535)	<.001
Total costs^b^	11 977 (7138-21 442)	9728 (6130-16 790)	17 496 (10 863-30 619)	<.001	18 779 (11 997-31 263)	16 451 (10 928-26 722)	20 893 (13 603-34 301)	<.001

Abbreviations: CDC, Centers for Disease Control and Prevention; DIC, disseminated intravascular coagulation; LOS, length of stay; NA, not applicable; PAS, placenta accreta spectrum; SM, surgical morbidity; SMM, severe maternal morbidity.

^a^ Total charges were charges converted to 2019 US dollars using annual consumer price indexes.

^b^ Total costs were charges converted to 2019 US dollars and adjusted using the Healthcare Cost and Utilization Project cost-to-charges ratio.^31^

When we restricted the analysis to those who underwent hysterectomy (n = 1475), patients with placenta previa (n = 786) vs without placenta previa (n = 689) continued to have significantly higher rates of surgical morbidity excluding hysterectomy (643 patients [81.8%] vs 506 patients [73.4%]), surgical morbidity excluding hysterectomy and/or transfusion (600 patients [76.3%] vs 421 patients [61.1%]), and hemorrhage (538 patients [68.4%] vs 360 patients [52.2%]; *P* < .001) as well as higher receipt of surgical procedures (eg, cesarean delivery: 777 patients [98.9%] vs 597 patients [86.6%]; *P* < .001) and higher resource use (total charges: median [IQR], $81 814 [$50 629-$144 535]) vs $64 578 [$40 300-$109 100]; *P* < .001; total costs: median [IQR], $20 893 [$13 603-$34 301]) vs $16 451 [$10 928-$26 722]; *P* < .001) (Table 2). Among those with vs without placenta previa who underwent hysterectomy, the differences in SMMs excluding hysterectomy (365 patients [46.4%] vs 330 patients [47.9%]; *P* = .60) and SMMs excluding transfusion and/or hysterectomy (133 patients [16.9%] vs 115 patients [16.7%]; *P* = .94) were no longer statistically significant, nor were the differences in blood product transfusion (286 patients [36.4%] vs 273 patients [39.6%]; *P* = .22), shock (63 patients [8.0%] vs 61 patients [8.9%]; *P* = .57), acute kidney injury (13 patients [1.7%] vs 16 patients [2.3%]; *P* = .45), disseminated intravascular coagulation or other coagulopathy (57 patients [7.3%] vs 59 patients [8.6%]; *P* = .38), urinary tract injury (33 patients [4.2%] vs 32 patients [4.6%]; *P* = .70), or receipt of oophorectomy (54 patients [6.9%] vs 58 patients [8.4%]; *P* = .28). Results of additional sensitivity analyses are shown in eTable 10 and eTable 11 in the Supplement.

### Association of Placenta Previa With Risk of Peripartum Outcomes

In the entire sample of patients with PAS, placenta previa was independently associated with a higher risk of study-defined surgical morbidities (aRR, 1.18; 95% CI, 1.13-1.23) and Centers for Disease Control and Prevention–defined SMMs (aRR, 1.19; 95% CI, 1.12-1.27) after adjusting for the variables in the predefined adjustment models (Table 3). In addition to cesarean delivery (aRR, 1.15; 95% CI, 1.12-1.17), placenta previa was associated with higher receipt of intra-arterial balloon occlusion (aRR, 1.78; 95% CI, 1.30-2.45), hysterectomy (aRR, 1.33; 95% CI, 1.23-1.44), and urinary system repair and cystectomy (aRR, 1.55; 95% CI, 1.16-2.08). With regard to specific outcomes, patients with placenta previa had a 1.44-fold (95% CI, 1.35-1.54) higher risk of experiencing peripartum hemorrhage. Patients with placenta previa also had a significantly higher risk of having a hospital LOS of 6 days or more (aRR, 1.38; 95% CI, 1.25-1.52) and adjusted total hospital costs of $21 000 or greater (aRR, 1.28; 95% CI, 1.15-1.43) (Table 3).

**Table 3.** Multivariable Poisson Regression Analysis of Association Between Placenta Previa and Risk of Maternal Outcomes in Patients With PAS Disorder

Outcome	All patients with PAS (N = 3793)		Patients with PAS who received hysterectomy (n = 1475)	
	RR (95% CI)	aRR (95% CI)^a^	RR (95% CI)	aRR (95% CI)^a^
Study-defined SM				
Any	1.30 (1.26-1.35)	1.18 (1.13-1.23)	NA	NA
Excluding transfusion	1.32 (1.28-1.37)	1.20 (1.15-1.25)	NA	NA
Excluding hysterectomy	1.29 (1.23-1.35)	1.25 (1.19-1.32)	1.12 (1.06-1.18)	1.16 (1.09-1.23)
Excluding transfusion and/or hysterectomy	1.34 (1.27-1.40)	1.36 (1.28-1.44)	1.25 (1.17-1.35)	1.32 (1.22-1.43)
CDC-defined SMM				
Any	1.58 (1.49-1.68)	1.19 (1.12-1.27)	NA	NA
Excluding transfusion	1.90 (1.76-2.05)	1.27 (1.18-1.37)	NA	NA
Excluding hysterectomy	1.30 (1.19-1.42)	1.12 (1.02-1.24)	1.00 (0.90-1.11)	1.04 (0.93-1.16)
Excluding transfusion and/or hysterectomy	1.45 (1.20-1.74)	1.18 (0.96-1.46)	1.02 (0.81-1.27)	1.10 (0.86-1.40)
Surgical procedure use				
Cesarean delivery	1.34 (1.31-1.38)	1.15 (1.12-1.17)	1.14 (1.11-1.18)	1.09 (1.06-1.11)
Hysterectomy	2.08 (1.91-2.25)	1.33 (1.23-1.44)	NA	NA
Oophorectomy	1.47 (1.06-2.04)	1.02 (0.71-1.45)	0.81 (0.57-1.16)	0.81 (0.55-1.20)
Cystoscopy	2.63 (2.13-3.24)	1.39 (1.16-1.67)	1.45 (1.18-1.77)	1.18 (0.97-1.42)
Urinary system repair and cystectomy	2.96 (2.30-3.82)	1.55 (1.16-2.08)	1.40 (1.07-1.83)	1.20 (0.90-1.60)
Intra-arterial balloon occlusion	2.74 (1.92-3.91)	1.78 (1.30-2.45)	2.05 (1.39-3.01)	1.64 (1.12-2.42)
Specific outcomes				
Hemorrhage	1.35 (1.27-1.42)	1.44 (1.35-1.54)	1.32 (1.22-1.44)	1.44 (1.31-1.58)
Blood products transfusion	1.25 (1.13-1.38)	1.11 (0.99-1.24)	0.98 (0.87-1.11)	1.03 (0.90-1.17)
Shock	1.44 (1.09-1.90)	1.18 (0.87-1.61)	0.91 (0.66-1.26)	1.04 (0.74-1.46)
Acute kidney injury	1.11 (0.60-2.07)	0.87 (0.42-1.80)	0.71 (0.35-1.48)	0.63 (0.27-1.47)
DIC or other coagulopathy	1.36 (1.01-1.84)	1.07 (0.77-1.48)	0.83 (0.57-1.19)	0.95 (0.66-1.37)
Urinary tract injury	2.01 (1.31-3.09)	1.22 (0.76-1.97)	0.93 (0.57-1.50)	0.87 (0.52-1.44)
Hospital LOS ≥6 d	2.52 (2.26-2.81)	1.38 (1.25-1.52)	1.62 (1.42-1.84)	1.24 (1.10-1.40)
Total cost ≥$21 000^b^	2.10 (1.86-2.36)	1.28 (1.15-1.43)	1.35 (1.19-1.53)	1.12 (0.99-1.27)

Abbreviations: aRR, adjusted risk ratio; CDC, Centers for Disease Control; DIC, disseminated intravascular coagulation; LOS, length of stay; NA, not applicable; PAS, placenta accreta spectrum; RR, risk ratio; SM, surgical morbidity; SMM, severe maternal morbidity.

^a^ Adjusted for age, data collection year, race and ethnicity, obesity status, Charlson Comorbidity Index score, PAS severity, previous cesarean delivery, multiparity, gestational weeks, use of assisted reproductive technology, multiple gestation, hospital bed size, and hospital teaching status.

^b^ Total cost was charges converted to 2019 US dollars and adjusted using the Healthcare Cost and Utilization Project cost-to-charges ratio.^31^

Among patients with PAS who underwent hysterectomy, placenta previa continued to be associated with a higher risk of surgical morbidities excluding hysterectomy (aRR, 1.16; 95% CI, 1.09-1.23), surgical morbidities excluding hysterectomy and/or transfusion (aRR, 1.32; 95% CI, 1.22-1.43), receipt of cesarean delivery (aRR, 1.09; 95% CI, 1.06-1.11), receipt of intra-arterial balloon occlusion (aRR, 1.64; 95% CI, 1.12-2.42), hemorrhage (aRR, 1.44; 95% CI, 1.31-1.58), longer hospital LOS (eg, ≥6 days: aRR, 1.24; 95% CI, 1.10-1.40), and higher inpatient costs eg, ≥$21 000: aRR, 1.12; 95% CI, 0.99-1.27) than no placenta previa (Table 3). However, although patients with placenta previa continued to have a higher risk of having SMMs (excluding transfusion and/or hysterectomy) ( aRR, 1.10; 95% CI, 0.86-1.40), a higher risk of receipt of cytoscopy (aRR, 1.18; 95% CI, 0.97-1.42) and urinary system repair and cystectomy (aRR, 1.20; 95% CI, 0.90-1.60), these results were not significant. In sensitivity analyses using different selection methods and definitions of collective outcomes, such as surgical morbidities or SMMs, most results for the adjusted association of placenta previa with the risk of peripartum outcomes were consistent between the predefined models and the stepwise models (eTable 12 and eTable 13 in the Supplement).

### Factors Associated With Placenta Previa Risk

To investigate the factors associated with the risk of placenta previa among patients with PAS, we used placenta previa as a dependent variable. This analysis revealed that Hispanic ethnicity (aRR, 1.25; 95% CI, 1.12-1.39), previous cesarean delivery (aRR, 1.75; 95% CI, 1.58-1.95), breech presentation (aRR, 1.18; 95% CI, 1.06-1.30), coexisting pulmonary hypertension (aRR, 1.68; 95% CI, 1.05-2.69), preexisting cardiac disease (aRR, 1.41; 95% CI, 1.17-1.72), gastrointestinal disease (aRR, 1.24; 95% CI, 1.12-1.36), and preexisting anemia (aRR, 1.32; 95% CI, 1.21-1.43) were associated with an increased risk of placenta previa complications (Table 4). Hypertensive disorder of pregnancy was associated with a decreased risk of placenta previa complications (aRR, 0.67; 95% CI, 0.46-0.96).

**Table 4.** Multivariable Poisson Regression Analysis of Factors Associated With Having Placenta Previa Complications in Patients With Placenta Accreta Spectrum Disorder (N = 3793)

Characteristic	RR (95% CI)	aRR (95% CI)
Demographic		
Age per 5-y increase	1.03 (0.99-1.07)	1.02 (0.98-1.07)
Race and ethnicity		
Black	1.07 (0.94-1.23)	0.97 (0.85-1.10)
Hispanic	1.39 (1.24-1.55)	1.25 (1.12-1.39)
White or missing	1 [Reference]	1 [Reference]
Other^a^	1.26 (1.09-1.44)	1.22 (1.07-1.39)
Obesity status	1.12 (1.04-1.20)	1.06 (0.99-1.14)
Current pregnancy status		
Previous cesarean delivery	1.92 (1.73-2.14)	1.75 (1.58-1.95)
Assisted reproductive technology	0.67 (0.38-1.17)	0.93 (0.56-1.55)
Multiple gestation	0.71 (0.54-0.95)	0.85 (0.64-1.11)
Gestational diabetes	1.10 (0.97-1.25)	1.06 (0.94-1.20)
Hypertensive disorder of pregnancy	0.52 (0.40-0.67)	0.67 (0.46-0.96)
Breech presentation	1.25 (1.11-1.40)	1.18 (1.06-1.30)
Obstetric comorbidities		
Pulmonary hypertension	1.83 (0.89-3.76)	1.68 (1.05-2.69)
Bleeding disorder, preexisting	1.22 (0.97-1.54)	1.18 (0.95-1.47)
Cardiac disease, preexisting	1.30 (1.04-1.63)	1.41 (1.17-1.72)
Chronic hypertension	0.87 (0.71-1.07)	0.84 (0.68-1.05)
Preeclampsia with severe features	0.55 (0.42-0.72)	0.76 (0.53-1.10)
Preeclampsia without severe features or gestational hypertension	0.52 (0.28-0.96)	0.92 (0.45-1.87)
Anemia, preexisting	1.45 (1.33-1.58)	1.32 (1.21-1.43)
Gastrointestinal disease	1.37 (1.24-1.52)	1.24 (1.12-1.36)
Thyrotoxicosis	1.46 (0.91-2.35)	1.47 (0.95-2.26)

Abbreviations: aRR, adjusted risk ratio; RR, risk ratio.

^a^ Other races and/or ethnicities included American Indian, Asian or Pacific Islander, and multiple or other races and/or ethnicities.

## Discussion

This cohort study found that, among patients with PAS disorders, placenta previa was independently associated with an increased risk of severe maternal and surgical morbidities, higher use of surgical procedures (including cesarean delivery, hysterectomy, cystoscopy, urinary system repair and cystectomy, and intra-arterial balloon occlusion), longer hospital LOS, and higher inpatient costs. Associations between placenta previa and a significantly higher risk of peripartum hemorrhage, blood product transfusion, shock, disseminated intravascular coagulation or other coagulopathy, and urinary tract injury were also found. Although fewer significant associations were found between placenta previa and severe maternal and surgical morbidities, surgical procedure use, hospital LOS, or inpatient costs among patients who underwent hysterectomy, a higher likelihood of hemorrhage among patients with PAS and placenta previa remained. Hypertensive disorder of pregnancy was associated with a decreased risk of placenta previa among patients with PAS.

The results of this study provide further data suggesting that placenta previa is a major risk factor associated with a higher likelihood of adverse maternal outcomes among patients with PAS. Placenta previa may not only be considered a risk factor for and screening sign of PAS, but it might also be viewed as an independent risk factor associated with an increased risk of worse maternal outcomes among patients with PAS. Care routines and procedures that target patients with placenta previa–complicated PAS may need to be assessed or refined to improve the outcomes of these patients.

Because findings from single-center and small-sample studies^28,30^ were inconsistent regarding the association between placenta previa and worse maternal outcomes, the present study included 3793 patients with PAS from a national multicenter database and found that placenta previa was an independent risk factor associated with overall severe maternal and surgical morbidities. Unlike previous studies that included only patients with PAS who had undergone cesarean delivery^4,51^ and/or hysterectomy,^52^ the current study included all patients with a PAS diagnosis and conducted sensitivity analyses among those who received cesarean delivery and hysterectomy. We also examined surgical procedure and resource use among patients with PAS who did and did not have placenta previa, which could inform both the clinical and financial aspects of risk.

This study also found that the risk of some maternal outcomes (eg, blood product transfusion, shock, and acute kidney injury) among patients with vs without placenta previa who underwent hysterectomy were not significantly different. The fact that patients with PAS without placenta previa may not have better maternal outcomes was observed in another study that included only patients with PAS who underwent hysterectomy,^30^ and this phenomenon was often associated with a low antepartum PAS diagnosis rate among patients without placenta previa. Carusi et al^30^ found that only 40 of 106 patients (38%) without placenta previa had antepartum PAS diagnoses compared with 213 of 245 patients (87%) with placenta previa. Prenatal diagnosis is important for PAS management because it can inform multidisciplinary care and surgical preparation. In this study, we were unable to determine based on *ICD-10* codes whether the patient had an antenatal PAS diagnosis or a prenatal PAS severity assessment. Future studies that include this diagnostic information could estimate the associations of a prenatal PAS diagnosis and multidisciplinary care with maternal outcomes among patients with suspected PAS.

### Strengths and Limitations

This study has several strengths. The study included a large contemporary cohort of 3793 patients with PAS from a national representative multicenter database. Because the incidence rate of PAS is generally lower than 1% to 2%,^2,3,4^ and previous studies investigating the association between placenta previa and PAS were mostly single-center studies based on samples of fewer than 400 patients,^28,29,30^ the large sample included in this study enabled the addition of more covariates to the multivariate models so that the adjusted association of placenta previa with patient outcomes could be estimated in a more unbiased manner. We also investigated maternal outcomes from different dimensions (eg, surgical and maternal morbidities), focusing on both integrated factors such as SMMs and specific outcomes such as transfusion or urinary tract injury, which provided a more comprehensive view of maternal outcomes among those with vs without placenta previa.

This study also has limitations. First, we relied on the coding precision of the NIS database because our exposures and outcomes were all extracted from the diagnosis and procedure codes in the database, which could have produced misclassification bias with regard to the study variables because we did not have the original patient records for validation.^53^ Nevertheless, we used a list of *ICD-10* codes that had been used in several other studies,^4,37,52^^,^ and we disclosed all the codes for data extraction (eTables 5-7 in the Supplement) to facilitate transparent reporting. Second, clinical information, such as the number of previous cesarean deliveries, body mass index, imaging features, operational setting (eg, surgical setting and surgeon specialty), blood loss volume, blood transfusion volume, and neonatal information, is currently unavailable in the NIS database. Future studies and multicenter cohorts may consider examining the association between placenta previa and PAS while taking these variables into consideration.

## Conclusions

In this cohort study, placenta previa complications in patients with PAS were associated with a higher risk of overall maternal and surgical morbidities and higher use of hysterectomy and cystoscopy, longer hospital LOS, and higher costs. According to current estimates, the incidence rate of PAS will increase to 1 in 200 women undergoing cesarean delivery by 2025. To improve the clinical outcomes of this severe maternal complication, care routines and procedures that aim to alleviate maternal and surgical morbidities in patients with placenta previa–complicated PAS are therefore needed, and the mechanisms underlying the simultaneous occurrence of multiple placental implantation abnormalities could be further explored.

## Notes

Supplement. **eTable 1.** Centers for Disease Control and Prevention (CDC)–Defined Severe Maternal Morbidities (SMMs) **eTable 2.** Previous Study–Defined Surgical Morbidities (SMs) **eTable 3.** *ICD-10* Codes for Specific Outcomes and Procedures **eTable 4.** *ICD-10* Codes for Placenta Accreta Spectrum (PAS) Disorders and Placenta Previa (PP) **eTable 5.** Demographic Characteristics and *ICD-10* Codes for Patients With Placenta Accreta Spectrum (PAS) Disorders **eTable 6.** *ICD-10-CM* Codes in Elixhauser and Charlson Comorbidity Indices **eTable 7.** *ICD-10-CM* Codes for Comorbidities in Maternal Comorbidity Score Proposed by Leonard et al **eTable 8.** Details of Model Design in Statistical Analysis Section **eTable 9.** Characteristics of Placenta Previa (PP) by Severity of Placenta Accreta Spectrum (PAS) **eTable 10.** Characteristics of Patients With Placenta Accreta Spectrum (PAS) Disorders With and Without Placenta Previa (PP) in the Invasive PAS and Cesarean Delivery Subgroups **eTable 11.** Maternal Outcomes of Placenta Previa (PP) and Non-PP Placenta Accreta Spectrum (PAS) Groups in the Invasive PAS and Cesarean Delivery Subgroups **eTable 12.** Multivariable Poisson Regression Analysis of Association Between Placenta Previa (PP) and Risk of Maternal Outcomes in Patients With Placenta Accreta Spectrum (PAS) Disorders (N = 3793) **eTable 13.** Multivariable Poisson Regression Analysis of Association Between Placenta Previa (PP) and Risk of Maternal Outcomes in Patients With Placenta Accreta Spectrum (PAS) Disorders Who Received Hysterectomy (n = 1475)
eReferences

## References

[1] Silver RM, Branch DW. Placenta accreta spectrum. N Engl J Med. 2018;378(16):1529-1536. doi:10.1056/NEJMcp1709324 29669225

[2] Jauniaux E, Bunce C, Grønbeck L, Langhoff-Roos J. Prevalence and main outcomes of placenta accreta spectrum: a systematic review and meta-analysis. Am J Obstet Gynecol. 2019;221(3):208-218. doi:10.1016/j.ajog.2019.01.233 30716286

[3] Wu S, Kocherginsky M, Hibbard JU. Abnormal placentation: twenty-year analysis. Am J Obstet Gynecol. 2005;192(5):1458-1461. doi:10.1016/j.ajog.2004.12.074 15902137

[4] Matsuzaki S, Mandelbaum RS, Sangara RN, . Trends, characteristics, and outcomes of placenta accreta spectrum: a national study in the United States. Am J Obstet Gynecol. 2021;225(5):534.e1-534.e38. doi:10.1016/j.ajog.2021.04.233 33894149

[5] Erfani H, Fox KA, Clark SL, . Maternal outcomes in unexpected placenta accreta spectrum disorders: single-center experience with a multidisciplinary team. Am J Obstet Gynecol. 2019;221(4):337.e1-337.e5. doi:10.1016/j.ajog.2019.05.035 31173748 PMC8651298

[6] D’Arpe S, Franceschetti S, Corosu R, . Emergency peripartum hysterectomy in a tertiary teaching hospital: a 14-year review. Arch Gynecol Obstet. 2015;291(4):841-847. doi:10.1007/s00404-014-3487-y 25253416

[7] de Gregorio A, Friedl TWP, Scholz C, Janni W, Ebner F, de Gregorio N. Emergency peripartal hysterectomy—a single-center analysis of the last 13 years at a tertiary perinatal care unit. J Perinat Med. 2019;47(2):169-175. doi:10.1515/jpm-2018-0149 30179854

[8] Shellhaas CS, Gilbert S, Landon MB, ; Eunice Kennedy Shriver National Institutes of Health and Human Development (NICHD) Maternal–Fetal Medicine Units Network (MFMU). The frequency and complication rates of hysterectomy accompanying cesarean delivery. Obstet Gynecol. 2009;114(2, pt 1):224-229. doi:10.1097/AOG.0b013e3181ad9442 19622981 PMC2771379

[9] van den Akker T, Brobbel C, Dekkers OM, Bloemenkamp KWM. Prevalence, indications, risk indicators, and outcomes of emergency peripartum hysterectomy worldwide: a systematic review and meta-analysis. Obstet Gynecol. 2016;128(6):1281-1294. doi:10.1097/AOG.0000000000001736 27824773

[10] Warshak CR, Ramos GA, Eskander R, . Effect of predelivery diagnosis in 99 consecutive cases of placenta accreta. Obstet Gynecol. 2010;115(1):65-69. doi:10.1097/AOG.0b013e3181c4f12a 20027036

[11] Clark SL, Belfort MA, Dildy GA, Herbst MA, Meyers JA, Hankins GD. Maternal death in the 21st century: causes, prevention, and relationship to cesarean delivery. Am J Obstet Gynecol. 2008;199(1):36.e1-36.e5. doi:10.1016/j.ajog.2008.03.007 18455140

[12] Fox KA, Shamshirsaz AA, Carusi D, . Conservative management of morbidly adherent placenta: expert review. Am J Obstet Gynecol. 2015;213(6):755-760. doi:10.1016/j.ajog.2015.04.034 25935779

[13] Shamshirsaz AA, Fox KA, Erfani H, . Multidisciplinary team learning in the management of the morbidly adherent placenta: outcome improvements over time. Am J Obstet Gynecol. 2017;216(6):612.e1-612.e5. doi:10.1016/j.ajog.2017.02.016 28213059

[14] Yan J, Chen D, Yang H. Expert opinion on placenta accreta spectrum disorders in China. Matern-Fetal Med*.* 2021;3(4):235-237. doi:10.1097/FM9.0000000000000126

[15] Shamshirsaz AA, Fox KA, Salmanian B, . Maternal morbidity in patients with morbidly adherent placenta treated with and without a standardized multidisciplinary approach. Am J Obstet Gynecol. 2015;212(2):218.e1-218.e9. doi:10.1016/j.ajog.2014.08.019 25173187

[16] Collins SL, Alemdar B, van Beekhuizen HJ, ; International Society for Abnormally Invasive Placenta (IS-AIP). Evidence-based guidelines for the management of abnormally invasive placenta: recommendations from the International Society for Abnormally Invasive Placenta. Am J Obstet Gynecol. 2019;220(6):511-526. doi:10.1016/j.ajog.2019.02.054 30849356

[17] American College of Obstetricians and Gynecologists; Society for Maternal-Fetal Medicine. Obstetric care consensus no. 7: placenta accreta spectrum. Obstet Gynecol. 2018;132(6):e259-e275. doi:10.1097/AOG.0000000000002983 30461695

[18] Imafuku H, Tanimura K, Shi Y, Uchida A, Deguchi M, Terai Y. Clinical factors associated with a placenta accreta spectrum. *Placenta*. 2021;112(9):180-184. doi:10.1016/j.placenta.2021.08.001 34375912

[19] Thurn L, Lindqvist PG, Jakobsson M, . Abnormally invasive placenta–prevalence, risk factors and antenatal suspicion: results from a large population-based pregnancy cohort study in the Nordic countries. BJOG. 2016;123(8):1348-1355. doi:10.1111/1471-0528.13547 26227006

[20] Bi S, Zhang L, Wang Z, . Effect of types of placenta previa on maternal and neonatal outcomes: a 10-year retrospective cohort study. Arch Gynecol Obstet. 2021;304(1):65-72. doi:10.1007/s00404-020-05912-9 33386958

[21] Barinov SV, Shmakov RG, Medyannikova IV, . Efficacy of distal haemostasis during caesarean delivery in women with placenta accreta spectrum disorders. *J Matern Fetal Neonatal Med*. Published online November 18, 2021. doi:10.1080/14767058.2021.2005019 34794371

[22] Jauniaux E, Kingdom JC, Silver RM. A comparison of recent guidelines in the diagnosis and management of placenta accreta spectrum disorders. Best Pract Res Clin Obstet Gynaecol. 2021;72:102-116. doi:10.1016/j.bpobgyn.2020.06.007 32698993

[23] Takeda S, Takeda J, Murayama Y. Placenta previa accreta spectrum: cesarean hysterectomy. Surg J (N Y). 2021;7(Suppl 1):S28-S37. doi:10.1055/s-0040-1721492 35036545 PMC8752195

[24] Oyelese Y, Smulian JC. Placenta previa, placenta accreta, and vasa previa. Obstet Gynecol. 2006;107(4):927-941. doi:10.1097/01.AOG.0000207559.15715.98 16582134

[25] Silver RM. Abnormal placentation: placenta previa, vasa previa, and placenta accreta. Obstet Gynecol. 2015;126(3):654-668. doi:10.1097/AOG.0000000000001005 26244528

[26] Jauniaux E, Moffett A, Burton GJ. Placental implantation disorders. Obstet Gynecol Clin North Am. 2020;47(1):117-132. doi:10.1016/j.ogc.2019.10.002 32008663

[27] Jauniaux E, Alfirevic Z, Bhide AG, ; Royal College of Obstetricians and Gynaecologists. Placenta praevia and placenta accreta: diagnosis and management: green-top guideline no. 27a. BJOG. 2019;126(1):e1-e48. doi:10.1111/1471-0528.15306 30260097

[28] Mulla BM, Weatherford R, Redhunt AM, . Hemorrhagic morbidity in placenta accreta spectrum with and without placenta previa. Arch Gynecol Obstet. 2019;300(6):1601-1606. doi:10.1007/s00404-019-05338-y 31691015 PMC6907737

[29] Heading R, Slade L, Kennedy-Andrews S, Atkinson E, Grivell R. A comparison of praevia and non-praevia outcomes in placenta accreta spectrum cases: a single centre analysis. Aust N Z J Obstet Gynaecol. Published online February 21, 2022. doi:10.1111/ajo.13491 35188274

[30] Carusi DA, Fox KA, Lyell DJ, . Placenta accreta spectrum without placenta previa. Obstet Gynecol. 2020;136(3):458-465. doi:10.1097/AOG.0000000000003970 32769646

[31] Healthcare Cost and Utilization Project. Cost-to-charge ratio files: user guide for National Inpatient Sample (NIS) CCRs. Agency for Healthcare Research and Quality. November 18, 2020. Accessed January 7, 2022. https://hcup-us.ahrq.gov/db/ccr/ip-ccr/CCR-NIS-UserGuide-2001-2018.pdf

[32] von Elm E, Altman DG, Egger M, Pocock SJ, Gøtzsche PC, Vandenbroucke JP; STROBE Initiative. The Strengthening the Reporting of Observational Studies in Epidemiology (STROBE) statement: guidelines for reporting observational studies. J Clin Epidemiol. 2008;61(4):344-349. doi:10.1016/j.jclinepi.2007.11.008 18313558

[33] Stanhope KK, Joseph NT, Platner M, . Validation of *ICD-10* codes for gestational and pregestational diabetes during pregnancy in a large, public hospital. Epidemiology. 2021;32(2):277-281. doi:10.1097/EDE.0000000000001311 33252439

[34] Centers for Disease Control and Prevention. How does CDC identify severe maternal morbidity? appendix 2: severe maternal morbidity indicators and corresponding *ICD-9-CM*/*ICD-10-CM*/PCS codes during delivery hospitalizations. Centers for Disease Control and Prevention. Updated December 26, 2019. Accessed January 7, 2022. https://www.cdc.gov/reproductivehealth/maternalinfanthealth/smm/severe-morbidity-ICD.htm

[35] Mandelbaum RS, Smith MB, Violette CJ, . Conservative surgery for ovarian torsion in young women: perioperative complications and national trends. BJOG. 2020;127(8):957-965. doi:10.1111/1471-0528.16179 32086987 PMC7772940

[36] Matsuo K, Mandelbaum RS, Nusbaum DJ, . National trends and outcomes of morbidly obese women who underwent inpatient hysterectomy for benign gynecological disease in the USA. Acta Obstet Gynecol Scand. 2021;100(3):459-470. doi:10.1111/aogs.14034 33111335

[37] Matsuo K, Youssefzadeh AC, Mandelbaum RS, . Hospital surgical volume–outcome relationship in caesarean hysterectomy for placenta accreta spectrum. BJOG. 2022;129(6):986-993. doi:10.1111/1471-0528.16993 34743389

[38] Tsai KY, Hsieh KY, Ou SY, Chou FHC, Chou YM. Comparison of Elixhauser and Charlson methods for discriminative performance in mortality risk in patients with schizophrenic disorders. Int J Environ Res Public Health. 2020;17(7):2450. doi:10.3390/ijerph17072450 32260241 PMC7177958

[39] Moore BJ, White S, Washington R, Coenen N, Elixhauser A. Identifying increased risk of readmission and in-hospital mortality using hospital administrative data: the AHRQ Elixhauser Comorbidity Index. Med Care. 2017;55(7):698-705. doi:10.1097/MLR.0000000000000735 28498196

[40] Leonard SA, Kennedy CJ, Carmichael SL, Lyell DJ, Main EK. An expanded obstetric comorbidity scoring system for predicting severe maternal morbidity. Obstet Gynecol. 2020;136(3):440-449. doi:10.1097/AOG.0000000000004022 32769656 PMC7523732

[41] Leonard SA, Main EK, Lyell DJ, . Obstetric comorbidity scores and disparities in severe maternal morbidity across marginalized groups. Am J Obstet Gynecol MFM. 2022;4(2):100530. doi:10.1016/j.ajogmf.2021.100530 34798329 PMC10980357

[42] Healthcare Cost and Utilization Project. Race—Race/Ethnicity of Patient. Agency for Healthcare Research and Quality. Updated September 17, 2008. Accessed July 27, 2022. http://www.hcup-us.ahrq.gov/db/vars/race/nisnote.jsp

[43] Terry AR, Jordan JT, Schwamm L, Plotkin SR. Increased risk of cerebrovascular disease among patients with neurofibromatosis type 1: population-based approach. Stroke. 2016;47(1):60-65. doi:10.1161/STROKEAHA.115.011406 26645253

[44] Han X, Jiang F, Needleman J, Zhou H, Yao C, Tang YL. Comorbidity combinations in schizophrenia inpatients and their associations with service utilization: a medical record–based analysis using association rule mining. Asian J Psychiatr. 2022;67:102927. doi:10.1016/j.ajp.2021.102927 34847493

[45] Guo Z, Han X, Zhang H, Zheng W, Yang H, Ma J. Association between pre-delivery coagulation indicators and invasive placenta accreta spectrum. Clin Appl Thromb Hemost. 2022;28:10760296211070580. doi:10.1177/10760296211070580 34994211 PMC8762652

[46] Guo Z, Han X, Zheng W, Yang H, Ma J. Placenta accreta spectrum among multiple gestation: a retrospective analysis based on a Chinese population. Front Endocrinol (Lausanne). 2022;13:862785. doi:10.3389/fendo.2022.862785 35663330 PMC9158523

[47] Guo Z, Ma J, Yang H. Is twin gestation an independent risk factor for placenta accreta spectrum? Am J Obstet Gynecol. 2022;226(3):446-447. doi:10.1016/j.ajog.2021.10.025 34699749

[48] Knol MJ, Le Cessie S, Algra A, Vandenbroucke JP, Groenwold RHH. Overestimation of risk ratios by odds ratios in trials and cohort studies: alternatives to logistic regression. CMAJ. 2012;184(8):895-899. doi:10.1503/cmaj.101715 22158397 PMC3348192

[49] Zou G. A modified Poisson regression approach to prospective studies with binary data. Am J Epidemiol. 2004;159(7):702-706. doi:10.1093/aje/kwh090 15033648

[50] Zou GY, Donner A. Extension of the modified Poisson regression model to prospective studies with correlated binary data. Stat Methods Med Res. 2013;22(6):661-670. doi:10.1177/0962280211427759 22072596

[51] Dang X, Zhang L, Bao Y, . Developing and validating nomogram to predict severe postpartum hemorrhage in women with placenta previa undergoing cesarean delivery: a multicenter retrospective case-control study. Front Med (Lausanne). 2022;8:789529. doi:10.3389/fmed.2021.789529 35223881 PMC8873861

[52] Matsuo K, Matsuzaki S, Vestal NL, . Utilizations and outcomes of intra-arterial balloon occlusion at cesarean hysterectomy for placenta accreta spectrum. Acta Obstet Gynecol Scand. 2021;100(12):2234-2243. doi:10.1111/aogs.14266 34622939

[53] Jotwani AR, Leonard SA, Butwick A, Lyell DJ. 842 Positive predictive value of *ICD-10* codes for placenta accreta syndrome: a single center validation study. Am J Obstet Gynecol. 2021;224(2)(suppl):S523-S524. doi:10.1016/j.ajog.2020.12.865

