# Supplementary material for: Association of Placenta Previa With Severe Maternal Morbidity Among Patients With Placenta Accreta Spectrum Disorder
Source: JAMA Netw Open. 2022 Aug 22;5(8):e2228002. doi: 10.1001/jamanetworkopen.2022.28002 (PMC9396360; doi:10.1001/jamanetworkopen.2022.28002)
Supplement: Supplement. — eTable 1. Centers for Disease Control and Prevention (CDC)–Defined Severe Maternal Morbidities (SMMs) eTable 2. Previous Study–Defined Surgical Morbidities (SMs) eTable 3. ICD-10 Codes for Specific Outcomes and Procedures eTable 4. ICD-10 Codes for Placenta Accreta Spectrum (PAS) Disorders and Placenta Previa (PP) eTable 5. Demographic Characteristics and ICD-10 Codes for Patients With Placenta Accreta Spectrum (PAS) Disorders eTable 6. ICD-10-CM Codes in Elixhauser and Charlson Comorbidity Indices eTable 7. ICD-10-CM Codes for Comorbidities in Maternal Comorbidity Score Proposed by Leonard et al eTable 8. Details of Model Design in Statistical Analysis Section eTable 9. Characteristics of Placenta Previa (PP) by Severity of Placenta Accreta Spectrum (PAS) eTable 10. Characteristics of Patients With Placenta Accreta Spectrum (PAS) Disorders With and Without Placenta Previa (PP) in the Invasive PAS and Cesarean Delivery Subgroups eTable 11. Maternal Outcomes of Placenta Previa (PP) and Non-PP Placenta Accreta Spectrum (PAS) Groups in the Invasive PAS and Cesarean Delivery Subgroups eTable 12. Multivariable Poisson Regression Analysis of Association Between Placenta Previa (PP) and Risk of Maternal Outcomes in Patients With Placenta Accreta Spectrum (PAS) Disorders (N = 3793) eTable 13. Multivariable Poisson Regression Analysis of Association Between Placenta Previa (PP) and Risk of Maternal Outcomes in Patients With Placenta Accreta Spectrum (PAS) Disorders Who Received Hysterectomy (n = 1475) eReferences [file jamanetwopen-e2228002-s001.pdf]

Supplementary Online Content

Han X, Guo Z, Yang X, Yang H, Ma J. Association of placenta previa with severe maternal morbidity among patients with placenta accreta spectrum disorder. *JAMA Netw Open*. 2022;5(8):e2228002. doi:10.1001/jamanetworkopen.2022.28002

eTable 1. Centers for Disease Control and Prevention (CDC)–Defined Severe Maternal Morbidities (SMMs)[1]

eTable 2. Previous Study–Defined Surgical Morbidities (SMs)

eTable 3. *ICD-10* Codes for Specific Outcomes and Procedures

eTable 4. *ICD-10* Codes for Placenta Accreta Spectrum (PAS) Disorders and Placenta Previa (PP)

eTable 5. Demographic Characteristics and *ICD-10* Codes for Patients With Placenta Accreta Spectrum (PAS) Disorders

eTable 6. *ICD-10-CM* Codes in Elixhauser and Charlson Comorbidity Indices

eTable 7. *ICD-10-CM* Codes for Comorbidities in Maternal Comorbidity Score Proposed by Leonard et al[3]

eTable 8. Details of Model Design in Statistical Analysis Section

eTable 9. Characteristics of Placenta Previa (PP) by Severity of Placenta Accreta Spectrum (PAS)

eTable 10. Characteristics of Patients With Placenta Accreta Spectrum (PAS) Disorders With and Without Placenta Previa (PP) in the Invasive PAS and Cesarean Delivery Subgroups

eTable 11. Maternal Outcomes of Placenta Previa (PP) and Non-PP Placenta Accreta Spectrum (PAS) Groups in the Invasive PAS and Cesarean Delivery Subgroups

eTable 12. Multivariable Poisson Regression Analysis of Association Between Placenta Previa (PP) and Risk of Maternal Outcomes in Patients With Placenta Accreta Spectrum (PAS) Disorders (N = 3793)

eTable 13. Multivariable Poisson Regression Analysis of Association Between Placenta Previa (PP) and Risk of Maternal Outcomes in Patients With Placenta Accreta Spectrum (PAS) Disorders Who Received Hysterectomy (n = 1475)

eReferences

This supplementary material has been provided by the authors to give readers additional information about their work.

© 2022 Han X et al. *JAMA Network Open*.

eTable 1. Centers for Disease Control and Prevention (CDC)–Defined Severe Maternal Morbidities (SMMs)

**Perioperative outcomes**	**Codes**
Acute myocardial infarction	I220, I221, I222, I228, I229, I2101, I2102, I2109, I2111, I2119, I2121, I2129, I213, I214, I219, I21A1, I21A9
Aneurysm	I711, I712, I713, I714, I715, I716, I718, I719, I790, I7100, I7101, I7102, I7103
Acute renal failure	N170, N171, N172, N178, N179, O904
Adult respiratory distress syndrome	J80, J951, J952, J953, R092, J9600, J9601, J9602, J9620, J9621, J9622, J95821, J95822
Amniotic fluid embolism	O8812, O8813, O88111, O88112, O88113
Cardiac arrest/ventricular fibrillation	I462, I468, I469, I4901, I4902
Conversion of cardiac rhythm	5A2204Z, 5A12012
Disseminated intravascular coagulation	D65, D688, D689, O723
Eclampsia	O151, O152, O159, O1500, O1502, O1503
Heart failure/arrest during surgery or procedure	I97120, I97121, I97130, I97131, I97710, I97711
Puerperal cerebrovascular disorders	I600, I601, I602, I603, I604, I605, I606, I607, I608, I609, I611, I612, I613, I614, I615, I616, I617, I618, I619, I620, I621, I629, I630, I631, I632, I633, I634, I635, I636, I638, I639, I650, I651, I652, I658, I659, I660, I661, I662, I668, I669, I670, I671, I672, I673, I674, I675, I676, I677, I678, I679, I680, I682, I688, O873, O2251, O2252, O2253, I97800, I97811, I97820, I97821
Pulmonary edema and Acute heart failure	J810, I501, I509, I5020, I5021, I5023, I5030, I5031, I5033, I5040, I5041, I5043
Severe anesthesia complications	O740, O741, O742, O743, O891, O892, O8901, O8909
Sepsis	O85, A327, A400, A401, A403, A408, A409, A419, A4101, A4102, A411, A412, A413, A414, A4150, A4151, A4152, A4153, A4159, A4181, A4189, O8604, R6520, T8144, T80211A, T814XXA, T8144XA, T8144XD, T8144XS
Shock	O751, R570, R571, R578, R579, R6521, T782XXA, T882XXA, T886XXA, T8110XA, T8111XA, T8119XA
Sickle cell disease with crisis	D5700, D5701, D5702, D57211, D57212, D57219, D57411, D57412, D57419, D57811, D57812, D57819

© 2022 Han X et al. *JAMA Network Open*.

Perioperative outcomes	Codes
Air and thrombotic embolism	I2601, I2602, I2609, I2690, I2692, I2699, 08802, O8803, O8822, O8823, O8832, O8833, O8881, O8882, O8883, O88111, O88112, O88113, O88114, O88115, O88116, O88117, O88118, O88119, O88211, O88212, O88213, O88214, O88215, O88216, O88217, O88218, O88219, O88311, O88312, O88313, O88314, O88315, O88316, O88317, O88318, O88319
Blood products transfusion	30233H1, 30233L1, 30233K1, 30233M1, 30233N1, 30233P1, 30233R1, 30233T1, 30233H0, 30233L0, 30233K0, 30233M0, 30233N0, 30233P0, 30233R0, 30233T0, 30230H1, 30230L1, 30230K1, 30230M1, 30230N1, 30230P1, 30230R1, 30230T1, 30230H0, 30230L0, 30230K0, 30230M0, 30230N0, 30230P0, 30230R0, 30230T0, 30240H1, 30240L1, 30240K1, 30240M1, 30240N1, 30240P1, 30240R1, 30240T1, 30240H0, 30240L0, 30240K0, 30240M0, 30240N0, 30240P0, 30240R0, 30240T0, 30243H1, 30243L1, 30243K1, 30243M1, 30243N1, 30243P1, 30243R1, 30243T1, 30243H0, 30243L0, 30243K0, 30243M0, 30243N0, 30243P0, 30243R0, 30243T0, 30250H1, 30250L1, 30250K1, 30250M1, 30250N1, 30250P1, 30250R1, 30250T1, 30250H0, 30250L0, 30250K0, 30250M0, 30250N0, 30250P0, 30250R0, 30250T0, 30253H1, 30253L1, 30253K1, 30253M1, 30253N1, 30253P1, 30253R1, 30253T1, 30253H0, 30253L0, 30253K0, 30253M0, 30253N0, 30253P0, 30253R0, 30253T0, 30260H1, 30260L1, 30260K1, 30260M1, 30260N1, 30260P1, 30260R1, 30260T1, 30260H0, 30260L0, 30260K0, 30260M0, 30260N0, 30260P0, 30260R0, 30260T0, 30263H1, 30263L1, 30263K1, 30263M1, 30263N1, 30263P1, 30263R1, 30263T1, 30263H0, 30263L0, 30263K0, 30263M0, 30263N0, 30263P0, 30263R0, 30263T0
Hysterectomy	0UT90ZZ, 0UT94ZZ, 0UT97ZZ, 0UT98ZZ, 0UT9FZZ
Temporary tracheostomy	0B110Z4, 0B110F4, 0B113Z4, 0B113F4, 0B114Z4, 0B114F4
Ventilation	5A1935Z, 5A1945Z, 5A1955Z

© 2022 Han X et al. *JAMA Network Open*.

eTable 2. Previous Study–Defined Surgical Morbidities (SMs)

**Peripartum outcomes**	**Codes**
Hemorrhage (antepartum, intrapartum, postpartum	O46, O72, O67, O441, O443, O445, R58
Chorioamnionitis or Endometritis	O411, O8612
Disseminated intravascular coagulopathy or other coagulopathy	D65, D684, D688, D689, R791, O723, O670, O4502, O4602
Systemic inflammatory syndrome or sepsis	A40, A41, O85, A021, A227, A267, A327, A427, B377, R651, R652, O0337, O0387, O0487, B377, R651, R652, O0337, O0387, O0487, O0737, O0882, O8604, T8144
Hypotension	I95, O265
Shock	R57, T882, T811, O751, T794, R6521, O2650, O0331, O0381, O0481, O0731
Pelvic hematoma	O717
Maternal distress	O750
Anesthesia complications	O29, O74, O89, T41, T882, T883, T885
Myocardial infarction	I21, I22, I23, I252, I9771
Respiratory failure	J96, R092, J9582
Pulmonary edema	J81, J681
Prolonged intubation	Z991
Pneumonia	J12, J13, J14, J15, J16, J17, J18, B012, B052, J100, J110, J842, J851, A0103, A0222, A3711, A3781, A3791, A5484, B0681, B7781, J09X1, J8281, J8282, J8411, J95851
Amniotic fluid embolism	O881
Venous thromboembolism or pulmonary embolism	I26, I81, I82, O032, O047, O082, O223, O882, O883, O888, I2724, I2782
Arterial embolism	I74, I75, I76
Cerebrovascular disease or stroke	I60, I61, I62, I63, I65, I66, I67, I68, I69 G45, G46, O873, I978
Acute kidney injury	N17, N19, N990, O904
Pyelonephritis	N10, N11, O230, O862, N137, A0225, D8684
Wound complications or abscess	L76, L02, L03, O860, O900, O902, T813, T814, N151, K681, K651
Urinary tract injury	S371, S372, N997, N9981

© 2022 Han X et al. *JAMA Network Open*.

eTable 3. *ICD-10* Codes for Specific Outcomes and Procedures

**Specific outcomes**	**Codes**
Hemorrhage (antepartum, intrapartum and postpartum)	O46, O72, O67, O441, O443, O445, R58
Blood products transfusion	30233H1, 30233L1, 30233K1, 30233M1, 30233N1, 30233P1, 30233R1, 30233T1, 30233H0, 30233L0, 30233K0, 30233M0, 30233N0, 30233P0, 30233R0, 30233T0, 30230H1, 30230L1, 30230K1, 30230M1, 30230N1, 30230P1, 30230R1, 30230T1, 30230H0, 30230L0, 30230K0, 30230M0, 30230N0, 30230P0, 30230R0, 30230T0, 30240H1, 30240L1, 30240K1, 30240M1, 30240N1, 30240P1, 30240R1, 30240T1, 30240H0, 30240L0, 30240K0, 30240M0, 30240N0, 30240P0, 30240R0, 30240T0, 30243H1, 30243L1, 30243K1, 30243M1, 30243N1, 30243P1, 30243R1, 30243T1, 30243H0, 30243L0, 30243K0, 30243M0, 30243N0, 30243P0, 30243R0, 30243T0, 30250H1, 30250L1, 30250K1, 30250M1, 30250N1, 30250P1, 30250R1, 30250T1, 30250H0, 30250L0, 30250K0, 30250M0, 30250N0, 30250P0, 30250R0, 30250T0, 30253H1, 30253L1, 30253K1, 30253M1, 30253N1, 30253P1, 30253R1, 30253T1, 30253H0, 30253L0, 30253K0, 30253M0, 30253N0, 30253P0, 30253R0, 30253T0, 30260H1, 30260L1, 30260K1, 30260M1, 30260N1, 30260P1, 30260R1, 30260T1, 30260H0, 30260L0, 30260K0, 30260M0, 30260N0, 30260P0, 30260R0, 30260T0, 30263H1, 30263L1, 30263K1, 30263M1, 30263N1, 30263P1, 30263R1, 30263T1, 30263H0, 30263L0, 30263K0, 30263M0, 30263N0, 30263P0, 30263R0, 30263T0
Shock	R57, T882, T811, O751, T794, R6521, O2650, O0331, O0381, O0481, O0731
Acute kidney injury	N17, N19, N990, O904
Disseminated intravascular coagulation or other coagulopathy	D65, D684, D688, D689, R791, O723, O670, O4502, O4602
Urinary tract injury	S371, S372, N997, N9981
**Surgical procedure use**	
Cesarean section	O82
Hysterectomy	0UB9, 0UT9, 0UT90ZL, 0UT97ZL, 0UT98ZL, 0UT9FZL
Oophorectomy	0UB0, 0UB1, 0UB2, 0UT0, 0UT1, 0UT2
Cystoscopy	0TJB7ZZ, 0TJB8ZZ, 0T768DZ, 0T778DZ, 0T788DZ
Urinary system repair & cystectomy	0TQB0ZZ, 0TQB7ZZ, 0TBB0ZZ, 0TTB0ZZ
Intra-arterial balloon occlusion	04LE3DT, 04LE3DZ, 04LE3ZT, 04LE3ZZ, 04LF3DU, 04LF3DZ, 04LF3ZU, 04LF3ZZ, 04LC3DZ, 04LC3ZZ, 04LD3DZ, 04LD3ZZ, 04L03DJ, 04L03DZ, 04L03ZZ

© 2022 Han X et al. *JAMA Network Open*.

eTable 4. *ICD-10* Codes for Placenta Accreta Spectrum (PAS) Disorders and Placenta Previa (PP)

ICD-10	Description
**ICD-10 codes for placenta accrete spectrum**	
O43211	Placenta accreta, first trimester
O43212	Placenta accreta, second trimester
O43213	Placenta accreta, third trimester
O43219	Placenta accreta, unspecified trimester
O43221	Placenta increta, first trimester
O43222	Placenta increta, second trimester
O43223	Placenta increta, third trimester
O43229	Placenta increta, unspecified trimester
O43231	Placenta percreta, first trimester
O43232	Placenta percreta, second trimester
O43233	Placenta percreta, third trimester
O43239	Placenta percreta, unspecified trimester
**ICD-10 codes for placenta previa**	
O4400	Complete placenta previa NOS or without hemorrhage, unspecified trimester
O4401	Complete placenta previa NOS or without hemorrhage, first trimester
O4402	Complete placenta previa NOS or without hemorrhage, second trimester
O4403	Complete placenta previa NOS or without hemorrhage, third trimester
O4410	Complete placenta previa with hemorrhage, unspecified trimester
O4411	Complete placenta previa with hemorrhage, first trimester
O4412	Complete placenta previa with hemorrhage, second trimester
O4413	Complete placenta previa with hemorrhage, third trimester
O4420	Partial placenta previa NOS or without hemorrhage, unspecified trimester
O4421	Partial placenta previa NOS or without hemorrhage, first trimester
O4422	Partial placenta previa NOS or without hemorrhage, second trimester
O4423	Partial placenta previa NOS or without hemorrhage, third trimester
O4430	Partial placenta previa with hemorrhage, unspecified trimester
O4431	Partial placenta previa with hemorrhage, first trimester
O4432	Partial placenta previa with hemorrhage, second trimester
O4433	Partial placenta previa with hemorrhage, third trimester
O4440	Low lying placenta NOS or without hemorrhage, unspecified trimester
O4441	Low lying placenta NOS or without hemorrhage, first trimester
O4442	Low lying placenta NOS or without hemorrhage, second trimester
O4443	Low lying placenta NOS or without hemorrhage, third trimester
O4450	Low lying placenta with hemorrhage, unspecified trimester
O4451	Low lying placenta with hemorrhage, first trimester
O4452	Low lying placenta with hemorrhage, second trimester
O4453	Low lying placenta with hemorrhage, third trimester

eTable 5. Demographic Characteristics and *ICD-10* Codes for Patients With Placenta Accreta Spectrum (PAS) Disorders

**Variables**	**ICD-10 codes**
**Pregnancy history**	
Grand multiparity	O094, Z641
Previous cesarean section	O3421, O3422, O6641
**Current pregnancy status**	
Assisted reproductive technology	O0981
Obesity	E660, E661, E662, E668, E669, Z683, Z684, O9921, E662, Z684, E6601
Tobacco use	F1721, F1722, F1729, O9933, T6522
PAS type	
Accreta	O4321
Invasive PAS	O4322, O4323
Increta	O4322
Percreta	O4323
Gestation weeks	
<27 weeks	Z3A26, Z3A25, Z3A24, Z3A23, Z3A22, Z3A21, Z3A20, Z3A19, Z3A18, Z3A17, Z3A16, Z3A15, Z3A14, Z3A13, Z3A12, Z3A11, Z3A10, Z3A9, Z3A8, Z3A7
28-31 weeks	Z3A31, Z3A31, Z3A30, Z3A29, Z3A28, Z3A27
32-33 weeks	Z3A32, Z3A33
34-36 weeks	Z3A34, Z3A35, Z3A36
>36 weeks	Z3A37, Z3A38, Z3A39, Z3A40, Z3A41, Z3A42, Z3A49
**Pregnancy complications**	
Preterm birth	Defined as <37 gestation weeks
Multiple gestation	O30, O31, O632, O661, Z372, Z373, Z374, Z375, Z376, O4302
Gestational diabetes mellitus	O11, O244
Hypertensive disorder of pregnancy	O14
Breech presentation	O321, O641
Preterm premature rupture of membrane	O4201, O4211, O4291
Fetal growth restriction	O365, Z364

© 2022 Han X et al. *JAMA Network Open*.

eTable 6. *ICD-10-CM* Codes in Elixhauser and Charlson Comorbidity Indices

**Elixhauser comorbidity index**	**ICD-10 codes**
Congestive heart failure	I09.9, I11.0, I13.0, I13.2, I25.5, I42.0, I42.5-I42.9, I43, I50, P29.0
Cardiac arrhythmias	I44.1-I44.3, I45.6, I45.9, I47-I49, R00.0, R00.1, R00.8, T82.1, Z45.0, Z95.0
Valvular disease	A52.0, I05-I08, I09.1, I09.8, I34-I39, Q23.0- Q23.3, Z95.2-Z95.4
Pulmonary circulation disorders	I26, I27, I28.0, I28.8, I28.9
Peripheral vascular disease	I70, I71, I73.1, I73.8, I73.9, I77.1, I79.0, I79.2, K55.1, K55.8, K55.9, Z95.8, Z95.9
Hypertension, uncomplicated	I10
Hypertension, complicated	I11-I13, I15
Paraplegia	G04.1, G11.4, G80.1, G80.2, G81, G82, G83.0-G83.4, G83.9
Other neurological disorders	G10-G13, G20-G22, G25.4, G25.5, G31.2, G31.8, G31.9, G32, G35-G37, G40, G41, G93.1, G93.4, R47.0, R56
Chronic pulmonary disease	I27.8, I27.9, J40-J47, J60-J67, J68.4, J70.1, J70.3
Diabetes without chronic complication	E10.0, E10.1, E10.9, E11.0, E11.1, E11.9, E12.0, E12.1, E12.9, E13.0, E13.1, E13.9, E14.0, E14.1, E14.9
Diabetes with chronic complication	E10.2-E10.8, E11.2-E11.8, E12.2-E12.8, E13.2-E13.8, E14.2- E14.8
Hypothyroidism	E00-E03, E89.0
Renal failure	I12.0, I13.1, N18, N19, N25.0, Z49.0- Z49.2, Z94.0, Z99.2
Liver disease	B18, I85, I86.4, I98.2, K70, K71.1, K71.3-K71.5, K71.7, K72-K74, K76.0, K76.2-K76.9, Z94.4
Peptic ulcer disease, excluding bleeding	K25.7, K25.9, K26.7, K26.9, K27.7, K27.9, K28.7, K28.9
AIDS/HIV	B20-B22, B24
Lymphoma	C81-C85, C88, C96, C90.0, C90.2
Metastatic cancer	C77-C80
Solid tumor without matastasis	C00-C26, C30- C34, C37-C41, C43, C45-C58, C60-C76, C97
Rheumatoid arthritis/collagen vascular diseases	L94.0, L94.1, L94.3, M05, M06, M08, M12.0, M12.3, M30, M31.0-M31.3, M32-M35
Coagulopathy	D65-D68, D69.1, D69.3-D69.6
Obesity	E66
Weight loss	E40-E46, R63.4, R64
Fluid and electrolyte disorders	E22.2, E86, E87
Blood loss anemia	D50.0

© 2022 Han X et al. *JAMA Network Open*.

**Elixhauser comorbidity index**	**ICD-10 codes**
Deficiency anemia	D50.8, D50.9, D51-D53
Alcohol abuse	F10, E52, G62.1, I42.6, K29.2, K70.0, K70.3, K70.9, T51, Z50.2, Z71.4, Z72.1
Drug abuse	F11-F16, F18, F19, Z71.5, Z72.2
Psychoses	F20, F22-F25, F28, F29, F30.2, F31.2, F31.5
Depression	F20.4, F31.3-F31.5, F32, F33, F34.1, F41.2, F43.2
**Charlson comorbidity index**	**ICD-10 codes**
Myocardial infarction	I21, I22, I25.2
Congestive heart failure	I09.9, I11.0, I13.0, I13.2, I25.2, I42.0, I42.5-I42.9, I43, I50, P29.0
Peripheral vascular disease	I70, I71, I73.1, I73.8, I73.9, I77.1, I79.0, I79.2, K55.1. K55.8, K55.9, Z95.8, K95.9
Cerebrovascular disease	G45, G46, H34.0, I60-169
Dementia	F00-F03, F05.1, G30, G31.1
Chronic pulmonary disease	I27.8, I27.9, J40-J47, J60-J67, J68.4, J70.1, J70.3
Rheumatic disease	M05, M06, M31.5, M32-M34, M35.1, M35.3, M36.0
Peptic ulcer disease	K25-K28
Mild liver disease	B18, K70.0-K70.3, K70.9, K71.3-K71.5, K71.7, K73, K74, K76.0, K76.2-K76.4, K76.8, K76.9, Z94.4
Diabetes without chronic complication	E10.0, E10.1, E10.6, E10.8-E11.1, E11.6, E11.8-E12.1, E12.6, E12.8-E13.1, E13.6, E13.8-E14.1, E14.6, E14.8, E14.9
Diabetes with chronic complication	E10.2-E10.5, E10.7, E11.2-E11.5, E11.7, E12.2-E12.5, E12.7, E13.2-E13.5, E13.7, E14.2-E14.5, E14.7
Hemiplegia or paraplegia	G04.1, G11.4, G80.2, G81, G82, G83.0-G83.4, G83.9
Renal disease	I12.0, I13.1, N03.2-N03.7, N05.2-N05.7, N18, N19, N25.0, Z49.0-Z49.2, Z94.0, Z99.2
Any malignancy, including lymphoma and leukemia, except malignant neoplasm of skin	C00-C26, C30-C34, C37-C41, C43, C45- C58, C60-C76, C81- C85, C88, C90-C97
Moderate or severe liver disease	I85.0, I85.9, I86.4, I98.2, K70.4, K71.1, K72.1, K72.9, K76.5, K76.6, K76.7
Metastatic solid tumor	C77-C80
AIDS/HIV	B20-B22, B24

© 2022 Han X et al. *JAMA Network Open*.

eTable 7. *ICD-10-CM* Codes for Comorbidities in Maternal Comorbidity Score Proposed by Leonard et al[2]

**Comorbidity Diagnosis Group**	**ICD-10 codes**
Gestational diabetes mellitus	O24.4
HIV/AIDS	O98.7, B20
Preexisting diabetes mellitus	E08-E13, O24.0, O24.1, O24.3, O24.8, O24.9, Z79.4
Previous cesarean birth	O34.21
Pulmonary hypertension	I27.0, I27.2
Twin/multiple pregnancy	O30, O31, Z37.2-Z37.7
Asthma, acute or moderate/severe (limited to moderate or severe asthma)	O99.5, J45.21, J45.22, J45.31, J45.32, J45.4, J45.5, J45.901, J45.902
Bleeding disorder, preexisting (added bleeding disorders--but not DIC or sickle cell with crisis)	D66-D69
BMI at delivery (categorized as ≥40)	Z68.4
Cardiac disease, preexisting (broader set of codes)	I05-I09, I11-I13, I15, I16, I20, I25, I27.8, I30-I41, I44-I49, I50.22, I50.23, I50.32, I50.33, I50.42, I50.43, I50.812, I50.813 O99.41, O99.42, Q20-Q24
Chronic hypertension	O10, O11, I10
Chronic renal disease (broader set of codes)	O26.83, I12, I13, N03- N05, N07, N08, N11.1, N11.8, N11.9, N18, N25.0, N25.1, N25.81, N25.89, N25.9, N26.9
Connective tissue or autoimmune disease (broad range of diseases that includes systemic lupus erythematosus)	M30-M36
Placenta previa, complete or partial	O44.03, O44.13, O44.23, O44.33
Preeclampsia with severe features (excludes eclampsia as eclampsia is included in SMM)	O14.1, O14.2, O11
Preeclampsia without severe features or gestational hypertension	O13, O14.0, O14.9
Substance use disorder (much broader set of codes)	F10-F19, O99.31, O99.32
Maternal age	Birth certificate (categorized as ≥35)
Anemia, preexisting (limited to codes that are likely present on delivery admission)	O99.01, O99.02, D50, D55, D56, D57.1, D57.20, D57.3, D57.40, D57.80, D55, D56, D58, D59
Bariatric surgery	O99.84
Gastrointestinal disease K (entire block),	O99.6, O26.6
Major mental health disorder	O99.34, F20-F39
Neuromuscular disease	O99.35, G40, G70
Placental abruption	O45
Placenta accreta spectrum	O43.2
Preterm birth (<37 weeks)	Z3A.20-Z3A.36
Thyrotoxicosis	E05

© 2022 Han X et al. *JAMA Network Open*.

eTable 8. Details of Model Design in Statistical Analysis Section

Model description	Dependent variable	Independent variables	Notes
Predefined adjustment models	Any SMM<br>Any SMM (exclude only transfusion)<br>Any SMM (exclude only hysterectomy)<br>Any SMM (exclude only transfusion & hysterectomy)<br>Any SM<br>Any SM (exclude only transfusion)<br>Any SM (exclude only hysterectomy)<br>Any SM (exclude only transfusion & hysterectomy)<br>Hemorrhage<br>Blood products transfusion<br>Shock<br>Acute kidney injury<br>DIC or other coagulopathy<br>Urinary tract injury<br>Cesarean section<br>Hysterectomy<br>Oophorectomy<br>Cystoscopy<br>Urinary system repair & cystectomy<br>Intra-arterial balloon occlusion<br>LOS ⩾ 6 days<br>Total adjusted cost ⩾ $ 21000	Exposure: PP<br>Adjusted for: age, data collection year, race (Black, Hispanic, Other, Unknown, White. (Other racial category included Asian, Pacific Islander, Native American)), obesity, CCI, PAS severity, previous cesarean delivery, multiparity, gestational weeks, the use of ART, multiple gestation, hospital bed size, hospital teaching status	1. Multivariable Poisson regression with robust error variance built in the general estimating equation (GEE) framework to control for the clustering effect of hospitals (exchangeable or the independent correlation were used as the within-group correlation structure in GEE models).<br>2. Since the hospital indicator in the NIS database can no longer be used to link hospital across years after the NIS redesign in 2012, we constructed a new hospital variable by combining the NIS hospital number and the year to identify hospitals.
Stepwise regression models	Any SMM	Exposure: PP	1. Stepwise logistic regressions

© 2022 Han X et al. *JAMA Network Open*.

Model description	Dependent variable	Independent variables	Notes
(Candidate variables)	Any SMM (exclude only transfusion)<br>Any SMM (exclude only hysterectomy)<br>Any SMM (exclude only transfusion & hysterectomy)<br>Any SM<br>Any SM (exclude only transfusion)<br>Any SM (exclude only hysterectomy)<br>Any SM (exclude only transfusion & hysterectomy)<br>Hemorrhage<br>Blood products transfusion<br>Shock<br>Acute kidney injury<br>DIC or other coagulopathy<br>Urinary tract injury<br>LOS ⩾ 6 days<br>Total adjusted cost ⩾ $ 21000	Adjusted for: age, data collection year, race, obesity, CCI, PAS severity, previous cesarean delivery, multiparity, gestational weeks, the use of ART, multiple gestation, hospital bed size, hospital teaching status, elective admission, gestational diabetes, breech, obesity, smoking, selected comorbidities from the 27 maternal comorbidity list, the use of cesarean delivery, hysterectomy, oophorectomy, cystoscopy, intra-arterial balloon occlusion as well as urinary system repair and cystectomy, hospital region, expected payer, patients estimated income quartile	(backward methods) were used on a pool of exposure variables to retain only the significant factors by setting the removal P value at 0.05.<br>2. If PP was excluded by the stepwise process, it would be manually entered into the final models.<br>3. The final models were fitted using multivariable Poisson regression with robust error variance built in the general estimating equation (GEE) framework to control for the clustering effect of hospitals (exchangeable or the independent correlation structures were used in the GEE models).
Stepwise regression models for surgical procedure use outcomes (Candidate variables)	Cesarean section<br>Hysterectomy<br>Oophorectomy<br>Cystoscopy<br>Urinary system repair & cystectomy<br>Intra-arterial balloon occlusion	Exposure: PP<br>Adjusted for: age, data collection year, race, obesity, smoking, previous cesarean delivery, multiparity, gestational weeks, the use of ART, multiple gestation, gestational diabetes, breech, obesity, smoking, selected comorbidities from the 27 maternal comorbidity list, hospital region, expected payer, patients estimated	Same as above.

© 2022 Han X et al. *JAMA Network Open*.

Model description	Dependent variable	Independent variables	Notes
		income quartile	
Multivariable Poisson regression on factors associated with being PP-complicated PAS patients (Candidate variables)	PP<br>(Patients with PP complication in the PAS population)	Age (per 5-year increase), data collection year, race, obesity, CCI, PAS severity, previous cesarean delivery, multiparity, the use of ART, multiple gestation, gestational diabetes, breech, obesity, selected comorbidities from the 27 maternal comorbidity list	We first conducted univariable Poisson regressions between being PP and each of the candidate variables. Factors with P value of 0.20 or less in the univariable models were then included in the multivariable model. The final models were fitted using multivariable Poisson regression with robust error variance built in the general estimating equation (GEE) framework to control for the clustering effect of hospitals (exchangeable or the independent correlation structures were used in the GEE models).
Details for sensitivity analyses:<br>1) Sensitivity analyses on the sets of adjusted covariates: Conducted using stepwise regressions reported above. Although we had a predefined adjustment model, we only included a minimum number of covariates and the adjusted variables were the same for all the outcomes. For the stepwise regression, the pool of candidate variables expanded and through stepwise variable selection, we might be able to adjust for different sets of variables in the adjustment model for different outcomes variables.<br>2) Sensitivity analyses on the sample: the predefined and stepwise models were conducted in both all the included PAS patients (N=3793) and in those who underwent hysterectomy (N=1475)			

© 2022 Han X et al. *JAMA Network Open*.

eTable 9. Characteristics of Placenta Previa (PP) by Severity of Placenta Accreta Spectrum (PAS)

Factor	Total (n = 1323)	Accreta (n = 918)	Increta (n = 181)	Percreta (n = 224)	*P* value
PP type					
Complete	1212 (91.6)	828 (90.2)	170 (93.9)	214 (95.5)	**.03**
Partial	45 (3.4)	40 (4.4)	NR[a]	NR[a]	
Low-lying	66 (5.0)	50 (5.5)	NR[a]	NR[a]	
PP with or without hemorrhage					
No or not specified	664 (50.2)	472 (51.4)	79 (43.6)	113 (50.4)	.16
With hemorrhage	659 (49.8)	446 (48.6)	102 (56.4)	111 (49.6)	
Trimester for PP diagnosis					
First trimester	NR[a]	NR[a]	NR[a]	NR[a]	**.04**
Second trimester	99 (7.5)	57 (6.2)	18 (9.9)	24 (10.7)	
Third trimester	1200 (90.7)	848 (92.4)	160 (88.4)	192 (85.7)	
Unspecified trimester	23 (1.7)	12 (1.3)	NR[a]	NR[a]	

NR, not reported.

[a] Data were suppressed for categories with fewer than 10 patients per requirements of the Healthcare Cost and Utilization Project.

Data are n (%) unless otherwise specified.

© 2022 Han X et al. *JAMA Network Open*.

eTable 10. Characteristics of Patients With Placenta Accreta Spectrum (PAS) Disorders With and Without Placenta Previa (PP) in the Invasive PAS and Cesarean Delivery Subgroups

	Invasive PAS (n = 768)				Cesarean section (n = 3079)				Hysterectomy (N=1475)			
	All (n = 768)	No PP (n = 363)	With PP (n = 405)	*P* value	All (n = 3079)	No PP (n = 1787)	With PP (n = 1292)	*P* value	All (n = 1475)	No PP (n = 689)	With PP (n = 786)	*P* value
**Demographics**												
Year												
2015 (4th quarter)	43 (5.6)	19 (5.2)	24 (5.9)	.54	177 (5.7)	97 (5.4)	80 (6.2)	.79	99 (6.7)	43 (6.2)	56 (7.1)	.12
2016	185 (24.1)	94 (25.9)	91 (22.5)		676 (22.0)	397 (22.2)	279 (21.6)		398 (27.0)	200 (29.0)	198 (25.2)	
2017	158 (20.6)	79 (21.8)	79 (19.5)		722 (23.4)	429 (24.0)	293 (22.7)		364 (24.7)	176 (25.5)	188 (23.9)	
2018	186 (24.2)	87 (24.0)	99 (24.4)		752 (24.4)	433 (24.2)	319 (24.7)		307 (20.8)	145 (21.0)	162 (20.6)	
2019	196 (25.5)	84 (23.1)	112 (27.7)		752 (24.4)	431 (24.1)	321 (24.8)		307 (20.8)	125 (18.1)	182 (23.2)	
Age at admission, median (IQR), y	33 (29, 37)	32 (29, 37)	33 (29, 37)	.42	33 (30, 37)	33 (30, 37)	33 (29, 37)	.35	33 (30, 37)	33 (30, 37)	33 (30, 37)	.45
Advanced maternal age (≥35y)	296 (38.5)	137 (37.7)	159 (39.3)	.67	1303 (42.3)	765 (42.8)	538 (41.6)	.52	621 (42.1)	300 (43.5)	321 (40.8)	.29
Race												
Black	142 (18.5)	77 (21.2)	65 (16.0)	.20	538 (17.5)	331 (18.5)	207 (16.0)	**<.001**	256 (17.4)	141 (20.5)	115 (14.6)	**.02**
Hispanic	181 (23.6)	78 (21.5)	103 (25.4)		671 (21.8)	342 (19.1)	329 (25.5)		380 (25.8)	161 (23.4)	219 (27.9)	
White	325 (42.3)	158 (43.5)	167 (41.2)		1367 (44.4)	834 (46.7)	533 (41.3)		607 (41.2)	287 (41.7)	320 (40.7)	

© 2022 Han X et al. *JAMA Network Open*.

	Invasive PAS (n = 768)				Cesarean section (n = 3079)				Hysterectomy (N=1475)			
	All (n = 768)	No PP (n = 363)	With PP (n = 405)	*P* value	All (n = 3079)	No PP (n = 1787)	With PP (n = 1292)	*P* value	All (n = 1475)	No PP (n = 689)	With PP (n = 786)	*P* value
[C] Other	87 (11.3)	35 (9.6)	52 (12.8)		365 (11.9)	198 (11.1)	167 (12.9)		167 (11.3)	75 (10.9)	92 (11.7)	
Unknown	33 (4.3)	15 (4.1)	18 (4.4)		138 (4.5)	82 (4.6)	56 (4.3)		65 (4.4)	25 (3.6)	40 (5.1)	
Elective admission	360 (46.9)	182 (50.1)	178 (44.0)	.10	1584 (51.4)	980 (54.8)	604 (46.7)	**<.001**	726 (49.2)	365 (53.0)	361 (45.9)	**.009**
Emergency department service use [b]	85 (11.1)	36 (9.9)	49 (12.1)	.34	335 (10.9)	179 (10.0)	156 (12.1)	.07	179 (12.1)	87 (12.6)	92 (11.7)	.59
Expected payers												
Medicare/Medicaid	441 (57.4)	202 (55.6)	239 (59.0)	.64	1449 (47.1)	789 (44.2)	660 (51.1)	**<.001**	794 (53.8)	372 (54.0)	422 (53.7)	.68
Private insurance	293 (38.2)	144 (39.7)	149 (36.8)		1452 (47.2)	891 (49.9)	561 (43.4)		607 (41.2)	279 (40.5)	328 (41.7)	
Other	34 (4.4)	17 (4.7)	17 (4.2)		178 (5.8)	107 (6.0)	71 (5.5)		74 (5.0)	38 (5.5)	36 (4.6)	
Hospital bed size												
Small	64 (8.3)	44 (12.1)	20 (4.9)	**.001**	343 (11.1)	229 (12.8)	114 (8.8)	**<.001**	141 (9.6)	82 (11.9)	59 (7.5)	**.02**
Medium	145 (18.9)	70 (19.3)	75 (18.5)		726 (23.6)	470 (26.3)	256 (19.8)		292 (19.8)	136 (19.7)	156 (19.8)	
Large	559 (72.8)	249 (68.6)	310 (76.5)		2010 (65.3)	1088 (60.9)	922 (71.4)		1042 (70.6)	471 (68.4)	571 (72.6)	
Hospital location/teaching status												
Rural	33 (4.3)	23 (6.3)	10 (2.5)	**<.001**	118 (3.8)	92 (5.1)	26 (2.0)	**<.001**	35 (2.4)	25 (3.6)	10 (1.3)	**<.001**
Urban	62 (8.1)	40 (11.0)	22 (5.4)		373	256	117 (9.1)		142 (9.6)	79 (11.5)	63 (8.0)	

© 2022 Han X et al. *JAMA Network Open*.

	Invasive PAS (n = 768)				Cesarean section (n = 3079)				Hysterectomy (N=1475)			
	All (n = 768)	No PP (n = 363)	With PP (n = 405)	*P* value	All (n = 3079)	No PP (n = 1787)	With PP (n = 1292)	*P* value	All (n = 1475)	No PP (n = 689)	With PP (n = 786)	*P* value
nonteaching					(12.1)	(14.3)						
Urban teaching	673 (87.6)	300 (82.6)	373 (92.1)		2588 (84.1)	1439 (80.5)	1149 (88.9)		1298 (88.0)	585 (84.9)	713 (90.7)	
Hospital region												
Northeast	134 (17.4)	61 (16.8)	73 (18.0)	.90	584 (19.0)	331 (18.5)	253 (19.6)	.22	276 (18.7)	140 (20.3)	136 (17.3)	.13
Midwest	166 (21.6)	76 (20.9)	90 (22.2)		604 (19.6)	338 (18.9)	266 (20.6)		294 (19.9)	128 (18.6)	166 (21.1)	
South	281 (36.6)	137 (37.7)	144 (35.6)		1123 (36.5)	679 (38.0)	444 (34.4)		537 (36.4)	262 (38.0)	275 (35.0)	
West	187 (24.3)	89 (24.5)	98 (24.2)		768 (24.9)	439 (24.6)	329 (25.5)		368 (24.9)	159 (23.1)	209 (26.6)	
**Pregnancy history**												
Grand multiparity	NR[a]	NR[a]	NR[a]	.81	36 (1.2)	23 (1.3)	13 (1.0)	.47	23 (1.6)	14 (2.0)	9 (1.1)	.17
Previous cesarean section	576 (75.0)	251 (69.1)	325 (80.2)	**<.001**	2089 (67.8)	1150 (64.4)	939 (72.7)	**<.001**	1082 (73.4)	462 (67.1)	620 (78.9)	**<.001**
**Current pregnancy status**												
Obesity												
Class 0 (Non-obesity)	607 (79.0)	295 (81.3)	312 (77.0)	.14	2544 (82.6)	1500 (83.9)	1044 (80.8)	**.02**	1183 (80.2)	570 (82.7)	613 (78.0)	**.02**
Class I-II	93 (12.1)	35 (9.6)	58 (14.3)		302 (9.8)	153 (8.6)	149 (11.5)		162 (11.0)	59 (8.6)	103 (13.1)	
Class III	68 (8.9)	33 (9.1)	35 (8.6)		233 (7.6)	134 (7.5)	99 (7.7)		130 (8.8)	60 (8.7)	70 (8.9)	
ART	NR[a]	NR[a]	NR[a]	.57	45 (1.5)	32 (1.8)	13 (1.0)	.07	17 (1.2)	13 (1.9)	4 (0.5)	**.01**
Charlson												

© 2022 Han X et al. *JAMA Network Open*.

	Invasive PAS (n = 768)				Cesarean section (n = 3079)				Hysterectomy (N=1475)			
	All (n = 768)	No PP (n = 363)	With PP (n = 405)	*P* value	All (n = 3079)	No PP (n = 1787)	With PP (n = 1292)	*P* value	All (n = 1475)	No PP (n = 689)	With PP (n = 786)	*P* value
comobidity index (CCI)												
=0	651 (84.8)	310 (85.4)	341 (84.2)	.43	2702 (87.8)	1569 (87.8)	1133 (87.7)	.65	1267 (85.9)	589 (85.5)	678 (86.3)	.73
=1	109 (14.2)	48 (13.2)	61 (15.1)		345 (11.2)	197 (11.0)	148 (11.5)		194 (13.2)	93 (13.5)	101 (12.8)	
=2	NR[a]	NR[a]	NR[a]		23 (0.7)	14 (0.8)	NR[a]		13 (0.9)	NR[a]	NR[a]	
>=3	NR[a]	NR[a]	NR[a]		NR[a]	NR[a]	NR[a]		NR[a]	NR[a]	NR[a]	
Elixhauser comorbidity index (ECI)												
=0	356 (46.4)	190 (52.3)	166 (41.0)	**.01**	1671 (54.3)	1031 (57.7)	640 (49.5)	**<.001**	676 (45.8)	333 (48.3)	343 (43.6)	.31
=1	240 (31.3)	101 (27.8)	139 (34.3)		880 (28.6)	485 (27.1)	395 (30.6)		472 (32.0)	210 (30.5)	262 (33.3)	
=2	116 (15.1)	51 (14.0)	65 (16.0)		377 (12.2)	199 (11.1)	178 (13.8)		232 (15.7)	106 (15.4)	126 (16.0)	
>=3	56 (7.3)	21 (5.8)	35 (8.6)		151 (4.9)	72 (4.0)	79 (6.1)		95 (6.4)	40 (5.8)	55 (7.0)	
Tobacco use	82 (10.7)	32 (8.8)	50 (12.3)	.11	255 (8.3)	140 (7.8)	115 (8.9)	.29	157 (10.6)	70 (10.2)	87 (11.1)	.57
PAS type												
Accreta	NA	NA	NA	.75	2364 (76.8)	1470 (82.3)	894 (69.2)	**<.001**	973 (66.0)	494 (71.7)	479 (60.9)	**<.001**
Increta	339 (44.1)	158 (43.5)	181 (44.7)		313 (10.2)	135 (7.6)	178 (13.8)		215 (14.6)	80 (11.6)	135 (17.2)	
Percreta	429	205	224		402	182	220		287	115	172	

© 2022 Han X et al. *JAMA Network Open*.

	Invasive PAS (n = 768)				Cesarean section (n = 3079)				Hysterectomy (N=1475)			
	All (n = 768)	No PP (n = 363)	With PP (n = 405)	*P* value	All (n = 3079)	No PP (n = 1787)	With PP (n = 1292)	*P* value	All (n = 1475)	No PP (n = 689)	With PP (n = 786)	*P* value
	(55.9)	(56.5)	(55.3)		(13.1)	(10.2)	(17.0)		(19.5)	(16.7)	(21.9)	
Trimester of PAS diagnosis												
First trimester	NR[a]	NR[a]	NR[a]	.10	NR[a]	NR[a]	NR[a]	.31	NR[a]	NR[a]	NR[a]	.90
Second trimester	66 (8.6)	28 (7.7)	38 (9.4)		159 (5.2)	82 (4.6)	77 (6.0)		117 (7.9)	57 (8.3)	60 (7.6)	
Third trimester	670 (87.2)	325 (89.5)	345 (85.2)		2808 (91.2)	1639 (91.7)	1169 (90.5)		1309 (88.7)	609 (88.4)	700 (89.1)	
Unspecified trimester	31 (4.0)	NR[a]	22 (5.4)		111 (3.6)	65 (3.6)	46 (3.6)		49 (3.3)	23 (3.3)	26 (3.3)	
Gestation weeks												
<32 weeks	157 (20.4)	52 (14.4)	105 (25.9)	**<.001**	414 (13.4)	172 (9.6)	242 (18.7)	**<.001**	274 (18.6)	91 (13.2)	183 (23.2)	**<.001**
32-33 weeks	120 (15.6)	39 (10.7)	81 (20.0)		343 (11.1)	129 (7.2)	214 (16.6)		210 (14.2)	72 (10.4)	138 (17.6)	
34-36 weeks	314 (40.9)	141 (38.8)	173 (42.7)		1133 (36.8)	528 (29.5)	605 (46.8)		640 (43.4)	274 (39.8)	366 (46.6)	
>36 weeks	148 (19.3)	125 (34.4)	23 (5.7)		1111 (36.1)	927 (51.9)	184 (14.2)		303 (20.5)	236 (34.3)	67 (8.5)	
Unknown	29 (3.8)	NR[a]	23 (5.7)		78 (2.5)	31 (1.7)	47 (3.6)		48 (3.3)	16 (2.3)	32 (4.1)	
**Pregnancy complications**												
Preterm birth	629 (81.9)	256 (70.5)	373 (92.1)	**<.001**	2254 (73.2)	1079 (60.4)	1175 (90.9)	**<.001**	1225 (83.1)	499 (72.4)	726 (92.4)	**<.001**
Multiple gestation	21 (2.7)	NR[a]	12 (3.0)	.68	141 (4.6)	104 (5.8)	37 (2.9)	**<.001**	44 (3.0)	24 (3.5)	20 (2.5)	.29
GDM	94 (12.2)	33 (9.1)	61 (15.1)	**.01**	406 (13.2)	236 (13.2)	170 (13.2)	.97	193 (13.1)	74 (10.7)	119 (15.1)	**.01**
HDP	30 (3.9)	17 (4.7)	13 (3.2)	.29	204 (6.6)	154 (8.6)	50 (3.9)	**<.001**	73 (4.9)	43 (6.2)	30 (3.8)	**.03**

© 2022 Han X et al. *JAMA Network Open*.

	Invasive PAS (n = 768)				Cesarean section (n = 3079)				Hysterectomy (N=1475)			
	All (n = 768)	No PP (n = 363)	With PP (n = 405)	*P* value	All (n = 3079)	No PP (n = 1787)	With PP (n = 1292)	*P* value	All (n = 1475)	No PP (n = 689)	With PP (n = 786)	*P* value
Breech presentation	141 (18.4)	64 (17.6)	77 (19.0)	.62	529 (17.2)	299 (16.7)	230 (17.8)	.44	272 (18.4)	118 (17.1)	154 (19.6)	.22
PPROM	47 (6.1)	28 (7.7)	19 (4.7)	.08	172 (5.6)	117 (6.5)	55 (4.3)	**.006**	90 (6.1)	55 (8.0)	35 (4.5)	**.005**
FGR	34 (4.4)	18 (5.0)	16 (4.0)	.50	165 (5.4)	112 (6.3)	53 (4.1)	**.008**	64 (4.3)	38 (5.5)	26 (3.3)	**.04**

PAS, placenta accreta spectrum; ART, assisted reproductive technology; GDM, gestational diabetes mellitus; HDP, hypertensive disorders of pregnancy; PPROM, preterm premature rupture of membranes; FGR, fetal growth restriction; IQR, interquartile range. Data are n (%) unless otherwise specified. NR, not reported.

[a] Data were suppressed for categories with fewer than 10 patients per requirements of the Healthcare Cost and Utilization Project.

[b] Records with evidence of emergency service use, per HCUP criteria.

[c] Other races and/or ethnicities included American Indian, Asian or Pacific Islander, and multiple or other races and/or ethnicities.

© 2022 Han X et al. *JAMA Network Open*.

eTable 11. Maternal Outcomes of Placenta Previa (PP) and Non-PP Placenta Accreta Spectrum (PAS) Groups in the Invasive PAS and Cesarean Delivery Subgroups

	Invasive PAS (N=768)				Cesarean section (N=3079)			
**Perioperative outcomes**	All (N=768)	Without PP (n=363)	With PP (n=405)	*P* value	All (N=3079)	Without PP (n=1787)	With PP (n=1292)	*P* value
**Study-defined surgical morbidities**								
Any	669 (87.1)	286 (78.8)	383 (94.6)	**<.001**	2323 (21.7)	1175 (65.8)	1148 (88.9)	**<.001**
Any (exclude only transfusion)	653 (85.0)	278 (76.6)	375 (92.6)	**<.001**	2255 (21.2)	1128 (63.1)	1127 (87.2)	**<.001**
Any (exclude only hysterectomy)	562 (73.2)	228 (62.8)	334 (82.5)	**<.001**	2008 (18.3)	1000 (56.0)	1008 (78.0)	**<.001**
Any (exclude transfusion and/or hysterectomy)	505 (65.8)	195 (53.7)	310 (76.5)	**<.001**	1815 (16.4)	870 (48.7)	945 (73.1)	**<.001**
**CDC-defined severe maternal morbidities**								
Any	586 (76.3)	248 (68.3)	338 (83.5)	**<.001**	1770 (19.0)	849 (47.5)	921 (71.3)	**<.001**
Any (exclude only transfusion)	533 (69.4)	214 (59.0)	319 (78.8)	**<.001**	1473 (17.3)	661 (37.0)	812 (62.8)	**<.001**
Any (exclude only hysterectomy)	334 (43.5)	146 (40.2)	188 (46.4)	.09	1034 (10.9)	529 (29.6)	505 (39.1)	**<.001**
Any (exclude transfusion and/or hysterectomy)	123 (16.0)	51 (14.0)	72 (17.8)	.17	319 (4.0)	153 (8.6)	166 (12.8)	**<.001**
**Specific outcomes**								
Hemorrhage	444 (57.8)	166 (45.7)	278 (68.6)	**<.001**	1614 (14.4)	751 (42.0)	863 (66.8)	**<.001**
Blood products transfusion	267 (34.8)	123 (33.9)	144 (35.6)	.65	857 (8.7)	448 (25.1)	409 (31.7)	**<.001**
Shock	59 (7.7)	25 (6.9)	34 (8.4)	.50	149 (1.9)	69 (3.9)	80 (6.2)	**.004**
Acute kidney injury	15 (2.0)	NR[a]	NR[a]	.61	31 (0.5)	15 (0.8)	16 (1.2)	.28
DIC or other coagulopathy	60 (7.8)	26 (7.2)	34 (8.4)	.59	154 (2.0)	80 (4.5)	74 (5.7)	.13
Urinary tract injury	25 (3.3)	NR[a]	17 (4.2)	.15	79 (0.8)	35 (2.0)	44 (3.4)	**.02**
**Surgical procedure use**								
Cesarean section	715 (93.1)	317 (87.3)	398 (98.3)	**<.001**	3079 (23.2)	1787 (100.0)	1292 (100.0)	NA

© 2022 Han X et al. *JAMA Network Open*.

	Invasive PAS (N=768)				Cesarean section (N=3079)			
**Perioperative outcomes**	All (N=768)	Without PP (n=363)	With PP (n=405)	*P* value	All (N=3079)	Without PP (n=1787)	With PP (n=1292)	*P* value
Hysterectomy	502 (65.4)	195 (53.7)	307 (75.8)	**<.001**	1374 (16.3)	597 (33.4)	777 (60.1)	**<.001**
Oophorectomy	56 (7.3)	27 (7.4)	29 (7.2)	.89	136 (1.8)	71 (4.0)	65 (5.0)	.18
Cystoscopy	218 (28.4)	76 (20.9)	142 (35.1)	**<.001**	476 (7.1)	178 (10.0)	298 (23.1)	**<.001**
Urinary system repair & cystectomy	132 (17.2)	45 (12.4)	87 (21.5)	**.001**	245 (4.3)	91 (5.1)	154 (11.9)	**<.001**
Intra-arterial balloon occlusion	89 (11.6)	30 (8.3)	59 (14.6)	**.007**	182 (2.9)	62 (3.5)	120 (9.3)	**<.001**
**Length of stay**								
Length of stay, median (IQR)	5 (4, 11)	4 (3, 6)	6 (4, 15)	**<.001**	4 (3, 7)	4 (3, 5)	5 (4, 11)	**<.001**
LOS after cesarean section, median (IQR)	4 (3, 5)	4 (3, 5)	4 (4, 5)	**<.001**	4 (3, 4)	3 (3, 4)	4 (3, 4)	**<.001**
**Charges**								
Total charges, median (IQR), USD (2019) [b]	79 935 (41 752, 136 043)	60 632 (30 197, 111 197)	96 898 (59 407, 164 408)	**<.001**	51 802 (29 909, 92 975)	41 338 (25 746, 72 455)	69 178 (41 662, 119 504)	**<.001**
Total costs, median (IQR), USD [c]	20 629 (11 584, 34 132)	15 071 (8 661, 27 746)	24 911 (16 186, 38 580)	**<.001**	13 179 (8 158, 22 659)	10 675 (6 946, 18 052)	17 445 (10 970, 30 619)	**<.001**

SM, surgical morbidity concept defined by the previous studies; SMM, severe maternal morbidity defined by the Centers for Disease Control and Prevention (CDC).

DIC, disseminated intravascular coagulation; LOS, length of stay; IQR, interquartile range. Data are n (%) unless otherwise specified. NR, not reported.

[a] Data were suppressed for categories with fewer than 10 patients per requirements of the Healthcare Cost and Utilization Project.

[b] Total charges were charges converted to 2019 US dollars using annual consumer price indexes. [c] Total costs were charges converted to 2019 US dollars and adjusted using the Healthcare Cost and Utilization Project cost-to-charges ratio.

© 2022 Han X et al. *JAMA Network Open*.

eTable 12. Multivariable Poisson Regression Analysis of Association Between Placenta Previa (PP) and Risk of Maternal Outcomes in Patients With Placenta Accreta Spectrum (PAS) Disorders (N = 3793)

Outcomes	RR (95% CI)	Predefined regression model * Adjusted RR (95% CI)	Stepwise regression model Adjusted RR (95% CI)
**Study-defined SM**			
Any	**1.30 (1.26-1.35)**	**1.18 (1.13-1.23)**	**1.16 (1.11-1.20)**
Any (exclude only transfusion)	**1.32 (1.28-1.37)**	**1.20 (1.15-1.25)**	**1.17 (1.12-1.22)**
Any (exclude only hysterectomy)	**1.29 (1.23-1.35)**	**1.25 (1.19-1.32)**	**1.23 (1.17-1.29)**
Any (exclude transfusion and/or hysterectomy)	**1.34 (1.27-1.40)**	**1.36 (1.28-1.44)**	**1.33 (1.26-1.41)**
**CDC-defined SMM**			
Any	**1.58 (1.49-1.68)**	**1.19 (1.12-1.27)**	**1.16 (1.09-1.23)**
Any (exclude only transfusion)	**1.90 (1.76-2.05)**	**1.27 (1.18-1.37)**	**1.22 (1.14-1.32)**
Any (exclude only hysterectomy)	**1.30 (1.19-1.42)**	**1.12 (1.02-1.24)**	1.08 (0.98-1.19)
Any (exclude transfusion and/or hysterectomy)	**1.45 (1.20-1.74)**	1.18 (0.96-1.46)	1.13 (0.92-1.38)
**Surgical procedure use**			
Caesarean section	**1.34 (1.31-1.38)**	**1.15 (1.12-1.17)**	**1.16 (1.13-1.19)**
Hysterectomy	**2.08 (1.91-2.25)**	**1.33 (1.23-1.44)**	**1.34 (1.24-1.45)**
Oophorectomy	**1.47 (1.06-2.04)**	1.02 (0.71-1.45)	1.02 (0.72-1.44)
Cystoscopy	**2.63 (2.13-3.24)**	**1.39 (1.16-1.67)**	**1.37 (1.13-1.66)**
Urinary system repair & cystectomy	**2.96 (2.30-3.82)**	**1.55 (1.16-2.08)**	**1.63 (1.22-2.18)**
Intra-arterial balloon occlusion	**2.74 (1.92-3.91)**	**1.78 (1.30-2.45)**	**1.62 (1.18-2.23)**
**Specific outcomes**			
Hemorrhage	**1.35 (1.27-1.42)**	**1.44 (1.35-1.54)**	**1.42 (1.33-1.51)**
Blood products transfusion	**1.25 (1.13-1.38)**	1.11 (0.99-1.24)	1.07 (0.97-1.19)
Shock	**1.44 (1.09-1.90)**	1.18 (0.87-1.61)	1.20 (0.87-1.64)
Acute kidney injury	1.11 (0.60-2.07)	0.87 (0.42-1.80)	0.97 (0.47-2.01)
DIC or other coagulopathy	**1.36 (1.01-1.84)**	1.07 (0.77-1.48)	1.03 (0.76-1.38)
Urinary tract injury	**2.01 (1.31-3.09)**	1.22 (0.76-1.97)	0.92 (0.61-1.40)
**Hospital LOS (≥ 6 days)**	**2.52 (2.26-2.81)**	**1.38 (1.25-1.52)**	**1.38 (1.25-1.52)**
**Total cost (≥ $21000)**	**2.10 (1.86-2.36)**	**1.28 (1.15-1.43)**	**1.18 (1.06-1.31)**

SM, surgical morbidity; CDC, Centers for Disease Control; SMM, severe maternal morbidity; DIC, disseminated intravascular coagulopathy; LOS, length of stay.
Statistically significant RR and 95% CI were presented in bold font.
* Adjusted for: age, data collection year, race, obesity, Charlson comorbidity index, PAS severity, previous cesarean delivery, multiparity, gestational weeks, the use of ART, multiple gestation, hospital bed size, hospital teaching status.

© 2022 Han X et al. *JAMA Network Open*.

eTable 13. Multivariable Poisson Regression Analysis of Association Between Placenta Previa (PP) and Risk of Maternal Outcomes in Patients With Placenta Accreta Spectrum (PAS) Disorders Who Received Hysterectomy (n = 1475)

Outcomes	RR (95% CI)	Predefined regression model * Adjusted RR (95% CI)	Stepwise regression model Adjusted RR (95% CI)
**Study-defined surgical SM**			
Any	NA	NA	NA
Any (exclude only transfusion)	NA	NA	NA
Any (exclude only hysterectomy)	**1.12 (1.06-1.18)**	**1.16 (1.09-1.23)**	**1.14 (1.07-1.20)**
Any (exclude only transfusion and/or hysterectomy)	**1.25 (1.17-1.35)**	**1.32 (1.22-1.43)**	**1.28 (1.20-1.38)**
**CDC-defined SMM**			
Any	NA	NA	NA
Any (exclude only transfusion)	NA	NA	NA
Any (exclude only hysterectomy)	1.00 (0.90-1.11)	1.04 (0.93-1.16)	1.01 (0.92-1.12)
Any (exclude only transfusion and/or hysterectomy)	1.02 (0.81-1.27)	1.1 (0.86-1.40)	1.10 (0.88-1.36)
**Surgical procedure use**			
Caesarean section	**1.14 (1.11-1.18)**	**1.09 (1.06-1.11)**	**1.09 (1.07-1.12)**
Hysterectomy	-	-	-
Oophorectomy	0.81 (0.57-1.16)	0.81 (0.55-1.20)	0.88 (0.62-1.26)
Cystoscopy	**1.45 (1.18-1.77)**	1.18 (0.97-1.42)	1.16 (0.96-1.41)
Urinary system repair & cystectomy	**1.40 (1.07-1.83)**	1.20 (0.90-1.60)	1.27 (0.98-1.66)
Intra-arterial balloon occlusion	**2.05 (1.39-3.01)**	**1.64 (1.12-2.42)**	**1.83 (1.10-3.05)**
**Specific outcomes**			
Hemorrhage	**1.32 (1.22-1.44)**	**1.44 (1.31-1.58)**	**1.41 (1.30-1.53)**
Blood products transfusion	0.98 (0.87-1.11)	1.03 (0.90-1.17)	1.03 (0.91-1.16)
Shock	0.91 (0.66-1.26)	1.04 (0.74-1.46)	1.06 (0.75-1.49)
Acute kidney injury	0.71 (0.35-1.48)	0.63 (0.27-1.47)	0.79 (0.33-1.87)
DIC or other coagulopathy	0.83 (0.57-1.19)	0.95 (0.66-1.37)	0.92 (0.68-1.25)
Urinary tract injury	0.93 (0.57-1.50)	0.87 (0.52-1.44)	0.82 (0.53-1.27)
**Hospital LOS (≥ 6 days)**	**1.62 (1.42-1.84)**	**1.24 (1.10-1.40)**	**1.22 (1.09-1.37)**
**Total cost (≥ $21000)**	**1.35 (1.19-1.53)**	1.12 (0.99-1.27)	1.05 (0.93-1.18)

SM, surgical morbidity; CDC, Centers for Disease Control; SMM, severe maternal morbidity; DIC, disseminated intravascular coagulopathy; LOS, length of stay; NA, not applicable.
Statistically significant RR and 95% CI were presented in bold font.
* Adjusted for: age, data collection year, race, obesity, Charlson comorbidity index, PAS severity, previous cesarean delivery, multiparity, gestational weeks, the

© 2022 Han X et al. *JAMA Network Open*.

use of ART, multiple gestation, hospital bed size, hospital teaching status.

© 2022 Han X et al. *JAMA Network Open*.

eReferences

1. Centers for Disease Control and Prevention. How does CDC identify severe maternal morbidity? appendix 2. severe maternal morbidity indicators and corresponding ICD-9-CM/ICD-10-CM/PCS codes during delivery hospitalizations. Centers for Disease Control and Prevention. Updated December 26, 2019. Accessed January 24, 2020. https://www.cdc.gov/reproductivehealth/maternalinfanthealth/smm/severe-morbidity-ICD.htm

2. Matsuzaki S, Mandelbaum RS, Sangara RN, et al. Trends, characteristics, and outcomes of placenta accreta spectrum: a national study in the United States. *Am J Obstet Gynecol*. 2021;225(5):534.e1-534.e38. doi:10.1016/j.ajog.2021.04.233

3. Leonard SA, Kennedy CJ, Carmichael SL, Lyell DJ, Main EK. An expanded obstetric comorbidity scoring system for predicting severe maternal morbidity. *Obstet Gynecol*. 2020;136(3):440-449. doi:10.1097/AOG.0000000000004022

© 2022 Han X et al. *JAMA Network Open*.
